# Nutrition Knowledge is Correlated with a Better Dietary Intake in Adolescent Soccer Players: A Cross-Sectional Study

**DOI:** 10.1155/2020/3519781

**Published:** 2020-01-03

**Authors:** Danilo C. Noronha, Monique I. A. F. Santos, Adrianny A. Santos, Lizia G. A. Corrente, Rúbia K. N. Fernandes, Anna C. A. Barreto, Ronyclay G. J. Santos, Rafaela S. Santos, Luís P. S. Gomes, Marcus V. S. Nascimento

**Affiliations:** ^1^Department of Nutrition, Tiradentes University, Murilo Dantas Avenue, 300 Farolândia, Aracaju 49032-971, Brazil; ^2^Biosciences Laboratory of Human Motricity, Tiradentes University, Murilo Dantas Avenue, 300 Farolândia, Aracaju 49032-971, Brazil

## Abstract

Nutrition education is one of the factors that may help to promote behavior change and therefore may improve the dietary habits of adolescent soccer players. However, information about the relationship between nutrition knowledge (NK) and the dietary behavior of these athletes is scarce. The purpose of this study was to evaluate the eating habits of adolescent soccer players and analyse the correlations among dietary intake and NK. Seventy-three Brazilian adolescent soccer players (aged 14–19 years), from four professional clubs, underwent anthropometric evaluation and completed 3-day food records. Misreporting of energy intake was evaluated and the dietary intake data were energy-adjusted and compared with recommendations for athletes and dietary reference intakes. The athletes also answered a questionnaire about barriers for healthy eating and a nutrition knowledge test divided into three sections: Basic Nutrition Knowledge (BNK), Sports Nutrition Knowledge (SNK), and Food Pyramid Nutrition Knowledge (FPNK). The participants showed a low NK (54.6%) and an inadequate intake of fruits, vegetables, dairy, carbohydrates, and micronutrients. A positive correlation was found between the ingestion of phosphorus and FPNK as well as among calcium and both SNK and Total NK (*p* < 0.05). Sodium intake was negatively correlated with all categories of the NK test (*p* < 0.05). The adolescents reported that the principal barriers for adopting a healthy diet were the lack of willpower and a busy lifestyle. In this context, nutrition education is recommended and should also provide practicable healthy eating goals according to athletes´ lifestyle as well as target motivational barriers to increase adherence.

## 1. Introduction

Nutrition is an important component contributing to the health and athletic performance of adolescent soccer players. The repeated high-intensity exercises such as running, brief sprints, tackling, and jumping activities can deplete fuel reserves, resulting in fatigue and reduced soccer performance [1]. Moreover, additional nutritional intake is also required to sustain optimal growth and development [2].

Despite the advantages of an appropriate diet, it seems, from the few studies available [2, 3], that young soccer athletes do not meet their nutritional goals. García-Rovés et al. [4] published the first systematic review about nutritional correlates of soccer players, which highlighted the need for further research in this area. The authors stated that there was little information about the intake food groups and related behaviors that accounted for the inadequate nutrient intake observed.

The lack of nutritional knowledge (NK) is considered one of the main causes of athletes´ inadequate dietary behavior [5]. Studies have reported that athletes have misconceptions about nutrition and are poorly informed about dietary guidelines, which may negatively influence their food choices [5, 6]. Although NK levels are typically reported, only a few studies analysed the relationship between NK and dietary intake in young athletes [5, 7]. This analysis is relevant since teenagers have lower nutrition knowledge than adults and, apparently, lack interest in nutrition [7]. In addition, this type of investigation may help to identify the benefits of improved NK and assist sport dietitians to better design nutrition education programs.

Even though there is a need to design and apply nutrition-specific interventions for adolescent soccer players, data about eating habits and other factors related to the dietary behavior of these athletes are scarce [4]. This information is crucial, as dietetic consultations should focus also on food, eating practice, and nutrition knowledge, not only on nutrients [4]. Therefore, the objective of the present study is to evaluate the eating habits of adolescent soccer players and analyse the correlations among dietary intake and nutrition knowledge.

## 2. Materials and Methods

### 2.1. Participants

Considering the standard deviation of previous studies using the same tool [7, 8], there would be necessary a total of 54 athletes to analyse the average nutrition knowledge in adolescent athletes at a 5% type I error with a precision of four percent in the percentage of correct responses. In this context, male adolescent soccer players (aged 14–19 years) were recruited from four professional clubs of the state of Sergipe, Brazil. Data were collected at the soccer training centres, during the preparation phase for the U-20 state level championship, which qualifies for the Brazilian Northeast Cup. To be eligible for the study, a participant had to be in preparation for the U-20 state level championship. The exclusion criteria were having any injury and a chronic or acute disease that would interfere with dietary habits. Additionally, vegans or lactose-intolerant athletes were not included.

Data collection occurred between October and December of 2016. Participants were recruited during training days and were told to arrive one hour before training to be evaluated. The athletes underwent anthropometric evaluation, completed 3-day food records, and answered a nutrition knowledge questionnaire. Assent and written consent were obtained from each of the participating soccer players and their families, respectively. All procedures involving human subjects were approved by the Research Ethics Committee of Tiradentes University (C. A. A. E 51660715.3.0000.5371).

### 2.2. Anthropometry and Body Composition

Anthropometric measures were performed following the techniques proposed by Lohman et al. [9]. Height was measured to the nearest 0.1 cm using a stadiometer (Altura Exata®, Altura Exata, Belo Horizonte, Brazil) and body weight was measured to the nearest 0.1 kg using an electronic scale (P150M®, LÍDER, Araçatuba, Brazil). All measurements were performed while the subjects wore no shoes and only light clothes (i.e., sleeveless T-shirt). Triceps and subscapular skinfolds were measured to the nearest 0.1 mm with a skinfold caliper (CESCORF®, CESCORF, Porto Alegre, Brazil). The stage of sexual maturation was obtained by the self-evaluation method, using the tables of Marshall and Tanner [10]. At that time, to our knowledge, there was no validated equation to calculate the body fat percentage of adolescent soccer players. Therefore, body fat percentage was estimated with the formula of Slaughter et al. [11] based on the triceps and subscapular skinfolds, as suggested by Rodriguez et al. [12].

The height/age index was used to diagnose growth delay. The growth curve of the World Health Organization (WHO) was used as reference [13].

### 2.3. Dietary Assessment

Participants recorded food intake for two nonconsecutive weekdays and one weekend day using a semistructured 3-day dietary record. Forms were provided and athletes were instructed by a sports dietitian to record all dietary information in as much detail as possible. All the reports were discussed with each athlete and the information was amended to include additional details as necessary. Athletes´ dietary intake was calculated by using the software DIETBOX 4.0 (Dietbox Informática LTDA-ME, Porto Alegre, Brazil). To increase data reliability, the energy and the nutrients were analysed hierarchically based upon the following nutrient databases: Brazilian Food Composition Table (TACO) [14], IBGE Food Composition Table [15], and the National Nutrient Database for Standard Reference from the United States Department of Agriculture [16]. For any processed foods for which the nutritional information was not available, the data were obtained from nutritional fact labels that were available on the food companies' websites.

### 2.4. Misreporting Evaluation

The basal metabolic rate (BMR) was estimated using the Schofield equation [17]. The Physical Activity Level (PAL) was estimated using the short version of the International Physical Activity Questionnaire (IPAQ), which was considered valid to evaluate the PAL in Brazilian adolescents [18]. Athletes were classified as moderate (PAL = 1.8) or vigorous (PAL = 2.0) using physical activity levels appropriated for adolescents [19]. The proportions of energy intake (EI) and the basal metabolic rate (EI : BMR) were compared by using the Goldberg cut-off points method [20], which was aimed at assessing the underreporting magnitude. Diet records with EIs below and above the cut-offs were considered underreporters and overreporters, respectively.

### 2.5. Nutrient and Food Servings Evaluation

The nutrient intake classification was performed after a comparison to specific recommendations [21]. Carbohydrate intake was compared to the recommendation of 5−7 g/kg·day^−1^, while to evaluate the protein ingestion, the recommendation of 1.2–2.0 g/kg·day^−1^ was used [20]. The World Health Organization guidelines [22] were used to evaluate the ingestion of total fat and the fatty acid compositions of the diets. In order to evaluate fiber intake, the cut-off point proposed by Williams et al. [23] was applied (chronological age plus five grams of fiber). Micronutrient intake was classified following the Institute of Medicine Dietary Reference Intakes [24]. The intakes of micronutrients under the Estimated Average Requirement (EAR) were considered inadequate. Since the mean intake of sodium in Brazil is elevated [25], the values of the Tolerable Upper Limit (UL) were used in order to determine the prevalence of individuals with an inadequate intake of this nutrient.

Food servings intake was compared to the recommendations proposed by the Brazilian Food Pyramid adapted to adolescent athletes [26]. Any food or energy-containing drink consumed within a 30-minute period was considered an “eating occasion” and the total number of eating occasions was compared to the recommendation proposed by Burke et al. [27].

### 2.6. Nutrition Knowledge Questionnaire

To evaluate nutrition knowledge, a two-part questionnaire was distributed to all the athletes. The first part of the questionnaire (questions 1–6) presented demographic questions, including age, hours of training/week, and perceived barriers to adopt a healthy diet [28].

The second part of the questionnaire consisted of a validated nutritional knowledge (NK) test [8]. The test had five questions (14 items) divided into three sections. The first section contained three multichoice questions about the basic aspects of nutrition (Basic Nutrition knowledge—BNK). In this section, the adolescents had to identify the best definition for healthy eating and the types of foods from the same food group and mark the best example of a balanced meal. The second part consisted of a question that was related to the Brazilian Food Guide Pyramid, where the athletes had to fill in the pyramid with the correct food groups (Food Pyramid Nutrition Knowledge—FPNK). The third section addressed the issue of sports nutrition (Sports Nutrition Knowledge—SNK) and it was comprised of 10 statements to which the athletes should mark “yes” if they agreed with the statement, “no” if they disagreed with the statement, or “do not know” if they were unsure. The sports nutrition section comprised questions about hydration, dietary supplements, type of foods that are beneficial for athletes, and pre- and posttraining meals. The correct issues were worth a plus point and the wrong or “do not know” answers received no points. The average percentage of correct answers was calculated.

The questionnaire had its discriminative validity determined in a previous study [8]. The test was applied to 19 graduates of the 4th year of nutrition and to 16 adolescent athletes. To be considered valid, the questionnaire had to be able to differentiate the participants at different levels of knowledge. After the application, the students had a significantly higher mean percentage of correct answers (97.4%) than did the athletes (57%).

### 2.7. Statistical Analyses

Data analysis was performed using SPSS Software Version 17.0 (SPSS Inc., Chicago, IL). Data normality was verified by the Kolmogorov-Smirnov test. Normally distributed data were presented as a mean and standard deviation (SD), while nonnormally distributed variables were log-transformed before statistical analyses. Categorical data were presented using absolute and relative prevalence. Due to underreporting, which is a common issue in the dietary assessment of adolescents [29, 30], data were energy-adjusted using the residual method. This approach was suggested by Poslusna et al. [31] as an alternative to handle misreporting, since exclusion of underreporters is not recommended [31, 32]. The residual method is done through the use of linear regression with total energy intake (EI) as the independent variable and intake of the nutrient of interest as the dependent variable. The energy-adjusted nutrient intake of each subject is determined by adding the residual—that is, the difference between the observed nutrient values for each subject and the values predicted from the regression equation—to the nutrient intake corresponding to mean EI of the study population [33]. This method decreases the influence of misreporting when interpreting results of studies based on food records.

The internal consistency of the nutritional knowledge questionnaire was obtained by Cronbach's alpha coefficient (*α*). A minimum value of 0.70 was recommended by Woodley [34]. Pearson's correlation coefficients between nutrition knowledge and dietary intake (food servings and nutrients) were calculated. The *r* values were interpreted as follows: values between 0 and 0.3 represented a weak correlation; values between 0.3 and 0.6 represented a moderate correlation; and values higher than 0.6 represented strong correlations [35]. For all analyses, a statistical significance was set at *p* < 0.05.

## 3. Results

### 3.1. Athletes' Characteristics and Anthropometric Data

All the seventy-three youth male soccer players of the four teams (age: 17.0 ± 1.3 years, years of experience in soccer: 7.4 ± 2.5, and hours of training/week: 12.1 ± 3.9) participated in the study. Participants were 1.82 ± 0.1 m tall and 66.4 ± 7 kg in mass. The athletes had an average body fat percentage of 14 ± 5 percent, 9.4 ± 4 kg of fat mass, and 56.9 ± 6 kg of lean mass. A total of 54 (74%) athletes were in the pubertal stage, 2 (2.7%) were prepubertal, and 17 (23.3%) were postpubertal. All athletes had an adequate height.

### 3.2. Dietary Intake

The prevalence of underreporting was 72.6% and there were no overreporters. Sixty-two adolescents (85%) have never been to a dietitian. Additionally, athletes showed a low number of eating occasions and low intakes of carbohydrates, unsaturated fats, fiber, cereals, fruits, vegetables, and dairy (Table 1). The participants also showed a high intake of oils and fats, sweets, meat, and eggs (Table 1).

**Figure 1:**
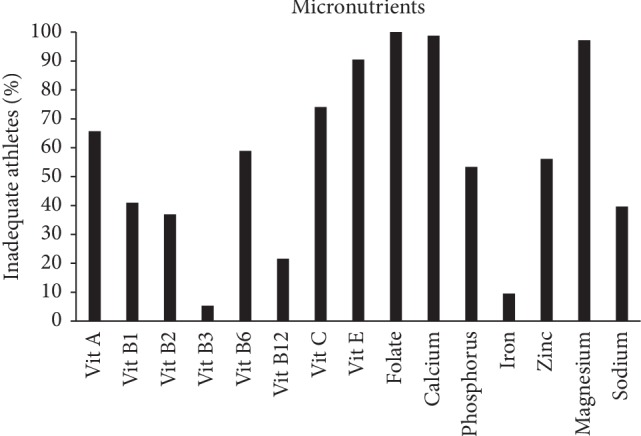
Prevalence of inadequacy in micronutrients (*n* = 73).

High prevalence of inadequacy was found in the intake of calcium, folate, magnesium, and vitamins C and E (Figure 1).

### 3.3. Nutrition Knowledge and Barriers for Healthy Eating

The internal consistency values of the nutritional knowledge questionnaire showed an acceptable reliability (0.82). Athletes responded correctly to approximately half of the nutrition knowledge questionnaire (score: 54.6 ± 13.6). The higher score was obtained in the BNK section (75.7 ± 22.6), followed by the SNK section (67.8 ± 21.1) and the FPNK section (16.3 ± 9.2).

The main findings regarding the questions of the NK test were that seventy-six percent of the participants reported that they did not know the Brazilian Food Pyramid, while approximately half of the athletes (45.8%) disagreed or did not know that it was possible to win a championship without using dietary supplements. Thirty-one adolescents (43%) referred to not knowing or incorrectly agreed with the statement “athletes should avoid potatoes and breads.” Moreover, 43% of the athletes were unaware of the fact or agreed that what an athlete eats was only important if he wants to gain or lose weight.

There was a significant negative correlation between the intake of sodium and Total NK, BNK, and FPNK (*p* < 0.05) (Table 2). There were a moderate positive correlation between the ingestion of phosphorus and FPNK (*p* < 0.05) and a weak positive correlation between the intake of calcium and total NK (*p* < 0.05) and SNK (*p* < 0.05) (Table 2).

**Figure 2:**
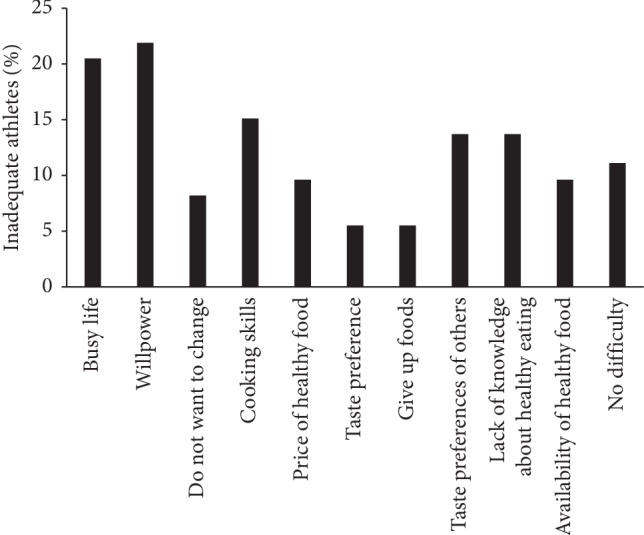
Athlete's barriers in trying to eat healthy (*n* = 73).

The main barriers for adopting a healthy diet reported by the athletes were the lack of willpower and busy lifestyle (Figure 2).

## 4. Discussion

This work analysed important aspects related to the dietary behavior of adolescent soccer players, such as barriers for healthy eating, nutrition knowledge, and food groups consumption. These factors should be taken into account during consultations and nutrition education programs. The results showed that the athletes' diet was poor and the main barriers for healthy eating were the lack of willpower and a busy lifestyle. Participants also showed low levels of NK; however, NK was positively correlated with a better dietary intake of some nutrients.

The prevalence of misreporting in the present study was 72.6%. Our finding is also consistent with the results of Vainik et al. [29] and Santos et al. [30], which showed prevalence of 66% and 74% of misreporting in adolescents, respectively. Therefore, the dietary data of the current work were energy-adjusted using the residual method, which is considered an appropriate tool to decrease the influence of misreporting in studies based on food records [31].

Carbohydrates are crucial for optimal performance in soccer, as muscle glycogen is the predominant substrate for energy production during a match. However, the athletes´ carbohydrate ingestion was also below the recommended levels. This finding is in agreement with a meta-analysis performed by Steffl et al. [36] regarding the macronutrient intake of soccer players. The authors suggested that soccer players may restrict carbohydrate as a consequence of warnings about carbohydrates overuse in the general population. In line with this, our hypothesis is that the low carbohydrate intake observed in the current study may be related to participants´ misconceptions about the role of carbohydrate in improving performance, since nearly 40% of the adolescents were unaware of the fact or incorrectly agreed that some easily accessible carbohydrate-rich foods in Brazil (potatoes and breads) should be avoided.

Additionally, the micronutrient ingestion of the participants was also inadequate, mainly due to the low consumption of dairy and FV and the high consumption of sweets and fats. This food pattern is common in Brazilian teenagers [37] and may result from increased autonomy in food choice [37]. A low consumption of FV may negatively influence the ingestion of indispensable nutrients, including folate and antioxidant vitamins (A, E, and C). Furthermore, inadequate consumption of dairy products may also affect the intake of bone-building nutrients, which are critical in achieving peak bone mass, maintaining skeletal integrity, and preventing fractures [21].

In general, the NK of the participants was low. Comparisons with other articles are difficult due to the low number of studies performed in adolescent athletes and the variety of methods applied. Nonetheless, to our knowledge, NK scores have ranged from 36.9% to 77.6% in teen athletes [7, 8, 37–43]. These studies have been performed in swimmers [37, 38], rugby players [39], soccer players [40], table tennis athletes [7], endurance athletes [41], and mixed populations of team and individual sports [8, 42–44]. It should be highlighted that the soccer players of the current work showed a nutrition knowledge similar to other adolescents of similar competitive level who were evaluated by Argôlo et al. [7] and Leite et al. [8] using the same questionnaire. In contrast, previous research by our group using the same tool showed a higher nutrition knowledge (73%) in high-level young athletes [41] than in the athletes of the present study. In these three articles [7, 8, 42], the lowest level of NK was also in the questions related to the Brazilian Food Pyramid. It seems that misconceptions about nutrition are more typical in regional or state-level competitors than in national/international athletes. Nonetheless, there is a lack of knowledge about how a healthy diet should be organized, regardless of competitive level. These findings highlight the need for nutrition education in adolescent athletes to stimulate the development of knowledge, skills, and autonomy in selecting a healthy diet.

There were a weak positive correlation between NK and the intake of bone-building nutrients and a weak negative correlation between all aspects of the NK test and sodium intake. There were no correlations between NK and food servings that could provide additional information about how this improved nutrient intake was translated into food habits. It appears that the analysis of food servings does not offer enough details of the dietary habits to detect such relationships, which may be represented by small reductions in the number of ultraprocessed/processed foods or little improvements in the intake of phosphorus and calcium-rich foods of different groups (e.g., milk and dark-green vegetables). This view is further supported by a systematic review published by Heaney et al. [6], which demonstrated that NK plays a small role in influencing food intake practices.

Another possible explanation for the weak correlations between nutrition knowledge and nutrient intake is lack of willpower, which was reported as a main barrier for healthy eating. A key component of inducing long-term behavior change is building sustained motivation, as individuals may develop their willpower only if they are motivated to do so [45, 46]. In this context, the science of health behavior change has increasingly emphasized theory-based approaches to intervention [47]. Motivational interventions based on self-determination theory or using motivational interviewing have focused on developing autonomous motivation (motivation associated with personal choice, interest, enjoyment, satisfaction, and value) instead of controlled motivation (motivated by pressures perceived by the individual or the attainment of external rewards) [47]. Being autonomously motivated may promote engagement in and maintenance of behaviors such as FV intake and other health-related behaviors among adolescents [46, 47]. In this context, future studies should test the efficacy of motivational interventions in athletes, as the more autonomously regulated an individual is toward a given behavior, the greater effort, engagement, persistence, and stability the individual is likely to evidence in that behavior [47, 48]. This may also help to overcome other barriers for healthy eating, such as the lack of time or cooking skills.

### 4.1. Limitations

Despite the relevance of this work, it has some limitations. Some players reported not having difficulties in adopting a healthy diet, which indicates that, as a consequence of their low NK level, some athletes might not know that they need to improve their food habits. Therefore, data about the barriers for healthy eating should be interpreted with caution. Another limitation is the underreporting prevalence observed. However, the energy-adjustment method applied improved the quality of the dietary data. It should also be noted that the adolescents of the present study were regional-level athletes and only a few of them had been to a dietitian before. From this perspective, the current findings should not be extrapolated to elite athletes, especially those who have access to a multidisciplinary team with dietitians.

### 4.2. Practical Applications

This research reveals some practical applications when consulting adolescent soccer athletes. Firstly, it is important for dietitians to evaluate overall dietary habits (e.g., number of eating occasions and types of food ingested) as this may provide practical information for nutrition advice and gives a better overview of what aspects of the diet should be improved. Secondly, it may be helpful to provide the athlete, verbally or using tools, explanations about sport nutrition and food guides, as they may have a lack of knowledge about these areas. Finally, since improvements in nutrition knowledge may not be completely translated in dietary behaviors, dietitians should discuss athletes' perceived barriers for healthy eating in order to increase adherence.

## 5. Conclusions

The athletes presented inadequate dietary habits and the main reported barriers for healthy eating were the lack of willpower and a busy lifestyle. NK levels were poor, especially about the Brazilian Food Pyramid. However, NK was positively correlated with a better dietary intake. In this context, to improve overall diet, nutrition education is recommended and should provide practicable healthy eating goals according to each athlete's lifestyle and target motivational barriers to increase adherence.

## Figures and Tables

**Table 1:** Dietary habits and macronutrient intake of the athletes (*n* = 73).

Dietary habits/macronutrients	Mean ± standard deviation	Recommendation
Number of eating occasions	3.7 ± 0.8	5 or more [26]
Macronutrients		
Carbohydrate (g/kg)	3.9 ± 1	5–7 [20]
Protein (g/kg)	1.4 ± 0.5	1.2–2.0 [20]
Fat (%)	25.1 ± 9.8	25–35 [20]
SFA (%)	8.5 ± 4.6	<10 [21]
MUFA (%)	8 ± 4.2	>10 [21]
PUFA (%)	3.5 ± 2	6–10 [21]
Fiber (g)	11.2 ± 6.5	Age + 5 g [22]
Food servings		
Cereals	5.2 ± 2.7	6–12 [25]
Fruits	2.8 ± 1.3	4–7 [25]
Vegetables	1.43 ± 0.7	5–7 [25]
Dairy	1.8 ± 0.9	2–4 [25]
Meat and eggs	4.2 ± 1.5	1, 5–3 [25]
Oils and fats	2.47 ± 1.2	1 [25]
Sweets	1.9 ± 1.3	1 [25]

SFA: saturated fatty acids; MUFA: monounsaturated fatty acids; PUFA: polyunsaturated fatty acids.

**Table 2:** Pearson's correlation coefficients among athlete's nutrition knowledge and dietary intake (*n* = 73).

Nutrition knowledge	Calcium	Phosphorus	Sodium
Total Nutrition Knowledge (TNK)	0.24^*∗*^	0.22	−0.24^*∗*^
Basic Nutrition Knowledge (BNK)	0.04	0.12	−0.25^*∗*^
Food Pyramid Nutrition Knowledge (FPNK)	0.18	0.39^*∗*^	−0.31^*∗*^
Sports Nutrition Knowledge (SNK)	0.28^*∗*^	0.08	−0.06^*∗*^

^*∗*^ *p* < 0.05.

## Data Availability

The SPSS data used to support the findings of this study are included within the supplementary information file.

## Supplementary Materials

Supplementary Materials The dataset of our research was provided as a Supplementary Material.
