# Supplementary material for: Nutrition Knowledge is Correlated with a Better Dietary Intake in Adolescent Soccer Players: A Cross-Sectional Study
Source: J Nutr Metab. 2020 Jan 3;2020:3519781. doi: 10.1155/2020/3519781 (PMC6964714; doi:10.1155/2020/3519781)
Supplement: Supplementary Materials — The dataset of our research was provided as a Supplementary Material. [file 3519781.f1.pdf]

NOME	numero_m	GÊNERO	TANNER	Classificaçã	Pubere_ou	FAIXA_ETÁ	PESO	ALTURA
L.E	5	1	4	2	1	17	65.3	1.77
L.C	4.666667	1	5	5	2	17	82	1.94
R.A	3.333333	1	2	2	1	16	73.2	1.75
J.P	3	1	3	2	1	15	59.8	1.72
Eugenio He	3	1	5	5	2	17	78	1.85
Olavo Sant	3	1	4	2	1	19	64.1	1.74
Helio Alves	3	1	4	2	1	16	62.6	1.7
V.S	2.666667	1	3	2	1	17	67	1.84
N.R.S	6	1	5	5	2	18	73.2	1.78
M.W	6	1	4	2	1	17	70.4	1.75
T.A	5	1	4	2	1	15	56.8	1.74
REGINALDO	4.666667	1	2	2	1	14	54.9	2
R.I	4.666667	1	4	2	1	19	59.3	1.74
Caio Freitas	4.666667	1	#NULL!	2	1	16	67.2	1.76
JUAN	4.333333	1	3	2	1	16	70.3	2
ADRIANO C	4.333333	1	5	5	2	19	66.8	1.75
A.R.S.	4.333333	1	3	2	1	16	63.3	1.73
IGOR DA CI	4	1	4	2	1	17	67.4	2
W.M	4	1	4	2	1	19	68.3	1.73
A.J	4	1	4	2	1	18	59.2	1.75
GUILHERM	4	1	5	5	2	20	70	1.74
L.N.J.	4	1	4	2	1	17	60.5	1.79
CAIQUE DE	4	1	4	2	1	17	67.2	1.79
ANDRE OLI	4	1	5	5	2	17	64.4	1.69
WANDERSO	4	1	3	2	1	18	64.1	1.73
ROMARIO A	3.666667	1	4	2	1	18	61.8	1.79
LEANDRO D	3.333333	1	5	5	2	16	57.7	1.69
JOSSUEL F.	3	1	3	2	1	18	64.2	2
CARLOS CE	3	1	1	1	1	17	54.8	2
EMERSON S	3	1	2	2	1	18	61.6	2
J.V	3	1	4	2	1	15	61.4	1.69
R.S	3	1	2	2	1	14	56.6	1.68
Mateus Cos	3	1	4	2	1	19	76.8	1.79
Thiago da S	3	1	9	5	2	18	71.4	1.78
Maxsuel Je	3	1	9	5	2	17	74	1.79
EVANIO DO	3	1	3	2	1	16	56.6	1.76
JACKSON M	3	1	3	2	1	16	57.4	1.69
L.O.	3	1	4	2	1	17	82.1	1.86
R.V.	3	1	5	5	2	19	75.3	1.79
E.F	2.666667	1	4	2	1	16	62.1	1.73
E.C	6	1	5	5	2	18	84.4	1.81
A.V	5.666667	1	1	1	1	17	61.1	1.76
D.A	4.666667	1	3	2	1	18	68.7	1.87
J.C.S.A.	4.333333	1	4	2	1	18	61.1	1.82
MATHEUS I	4	1	5	5	2	19	74.6	1.78
IAGO DOS S	4	1	3	2	1	18	68.4	1.79

Y.R	3.666667	1	4	2	1	16	67.5	1.69
RANYERES	3.666667	1	5	5	2	16	84.5	1.83
ANDERSON	3	1	3	2	1	16	57.5	2
ADONES S.	3	1	2	2	1	18	69	2
GUSTAVO J	3	1	3	2	1	17	60.2	2
S.H	3	1	4	2	1	16	74.1	1.7
HUDSON B.	3	1	4	2	1	16	52.8	1.74
IGOR FARIA	3	1	4	2	1	16	68.6	1.78
B.B.W	3	1	4	2	1	19	79.9	1.76
F.W	5	1	4	2	1	17	63.3	1.82
Felipe Gom	5	1	5	5	2	19	67.2	1.76
JOSE RICAR	3.666667	1	2	2	1	16	56.6	2
ALDO	3.666667	1	4	2	1	18	64.5	2
JOAO VICT(	3.666667	1	2	2	1	17	64	2
Erick Andre	3.666667	1	3	2	1	17	62.6	1.75
ADERLAN D	3.333333	1	5	5	2	15	75.2	2
V.L	3.333333	1	5	5	2	18	67.8	1.75
P.H	3.333333	1	4	2	1	16	78.1	1.76
J.C.M.	3.333333	1	4	2	1	17	58.3	1.68
YURE WEN	3	1	3	2	1	17	60.6	2
L.A	3	1	3	2	1	17	65.8	1.76
GERÔNIMC	3	1	4	2	1	19	66.4	1.84
GERSON LE	4	1	4	2	1	18	65.4	2
JOAO VITO	3.666667	1	4	2	1	14	54	2
PEDRO HEI	3.666667	1	5	5	2	18	72.2	2
FLAVIO S V.	3	1	2	2	1	17	71.2	2
ALEANDRO	3	1	3	2	1	15	75.9	2

@_GORD	MAS_MAG	MAS_GORI	IMC	CHO_KG	Perc_carbc	ADQ_CARB	MÉDIA_A	PTN_kg
13.91	56.21	9.08	20.84	3.12144	71.07043	3	36.08	0.552527
10.85	73.1	8.89	21.79	6.442308	72.967	3	55.52	0.677073
16.43	61.17	12.02	23.9	1.508333	47.71563	3	56.79667	0.775911
11.77	52.75	7.04	20.21	1.898829	22.75289	3	54.67	0.914214
6.63	72.82	5.1752	22.79	3.2738	51.16845	3	159.795	2.05
8.53	58.63	5.4659	21.17	2.6171	42.66895	3	68.193	1.06
8.84	57.06	5.5329	21.66	3.4702	36.02864	3	96.48	1.54
11.28	59.44	7.55	19.79	2.012836	18.4938	3	50.66	0.756119
12.76	63.86	9.33	23.1	4.519536	55.9998	3	102.575	1.401298
19.35	56.77	13.62	22.99	4.651207	63.36393	3	73.145	1.038991
9.35	51.48	5.31	18.76	4.28794	59.33275	3	101.235	1.782306
9.7	49.58	5	20	6.29	52	1	119	2.17
9.97	53.38	5.91	19.59	2.72552	45.50356	3	124.28	2.095784
14.5	57.46	9.74	#NULL!	4.69	106.5323	3	183.9333	2.73
15.79	59.2	11	23	7.1	56	2	121	1.73
14.6	47.04	9.75	21.57	4.92	61.96065	3	87.97	1.31
19.2	51.14	12.15	21.15	7.01	61.45221	1	74.94667	1.18
12.1	59.24	8	23	3.31	54	3	77	1.14
13.43	59.12	9.17	22.82	3.27694	57.89598	3	115.82	1.695754
7.9	54.52	4.67	19.33	5.445355	53.46497	1	76.09	1.285304
16.82	58.22	11.77	23.12	11.25	66.72566	2	187.79	2.68
10.98	53.85	6.64	18.88	5.61	57.87772	1	110.42	1.82
19.91	53.81	13.38	20.97	6.84	67.36281	1	106.26	1.58
12.29	56.48	7.91	22.55	3.34	45.73956	3	94.57	1.46
20.03	51.26	12.83	21.42	4.26	58.50615	3	85.48	1.33
16.79	51.42	10.37	19.29	4.45	50.5824	3	119.67	1.93
17.5	57.59	10.1	20.2	4.09	59.51597	3	62.33	1.08
14.86	54.66	10	24	3.64	50	3	134	2.08
12	48.92	7	21	4.65	51	3	93	1.69
9.46	55.77	6	21	4.08	48	3	102	1.65
17.25	50.81	10.58	21.5	2.745494	49.60568	3	97.09333	1.581325
8.53	51.77	4.82	20.05	2.645642	50.55139	3	85.75667	1.515135
8.53	70.25	6.5488	23.97	3.0565	60.72204	3	147.95	1.93
14.5	61	10.35	22.54	2.6209	35.82485	3	136.807	1.92
14.5	63.6	10.73	23.1	2.7941	84.80076	3	146.66	1.98
16.25	47.4	9.19	18.27	4.08	52.18	3	76.09	1.34
11.48	50.81	6.58	20.1	5.05	58.91553	1	94.28	1.64
24.66	65.63	16.46	23.73	2.64	45.55335	3	68.24	0.83
22.91	58.05	17.24	23.5	3.63	51.59448	3	23.95333	0.31
11.18	55.15	6.94	20.75	4.056361	64.0721	3	95.55333	1.538701
16.38	70.57	13.82	25.76	1.628495	50.35722	3	47.79	0.566232
10.64	54.59	6.5	19.72	5.38437	60.75439	1	82.195	1.345254
14.86	58.49	10.2	19.65	4.65182	66.50089	3	72.2	1.050946
19.31	49.29	11.8	18.45	4.91	56.64415	3	84.18667	1.37
17.13	61.82	12.77	23.55	4.84	52.47687	3	99.3	1.33
14.39	58.55	9.84	21.35	4.15	52.98776	3	129.06	1.88

20.78	53.47	14.02	23.63	2.594296	54.40466	3	63.825	0.945556
20.94	66.8	17.69	25.23	3.39	44.9486	3	127.7	1.51
8.11	52.83	5	19	4.3	55	3	67	1.16
14	59.34	10	21	3.49	41	3	146	2.12
16.06	50.53	10	19	3.67	52	3	87	1.44
18.04	60.73	13.36	25.64	7.983857	53.4951	3	99.63	1.344534
10.07	47.48	5.31	17.44	3.38	58.27824	3	50.01	0.94
16.25	57.45	11.14	21.65	4.53	74.91336	3	37.27	0.54
30.42	55.59	24.3	25.79	2.32	61.66089	3	49.90667	0.62
8.53	57.9	5.397	19.11	6.621564	57.47874	1	85.005	1.342891
14.5	57.46	9.74	#NULL!	5.06	83.01705	1	213.677	3.17
11.38	50.16	6	20	5.57	58	1	98	1.74
8.42	59.06	5	22	3.31	55	3	71	1.11
10.1	57.53	6	20	4.12	47	3	92	1.43
8.94	57	5.5976	20.44	3.8925	71.7042	3	94.96	1.52
18.73	61.11	14	26	3.35	60	3	78	1.03
12.76	59.15	8.64	22.14	2.632375	65.72243	3	54.71	0.806932
19.94	62.52	15.57	25.21	1.829257	58.59385	3	62.595	0.801472
9.55	52.73	5.56	20.66	3	42.23947	3	105.97	1.81
9.9	54.6	6	20	5.12	67	1	57	0.93
14.86	56.02	9.77	21.24	3.133359	60.65242	3	62.62	0.951672
12.46	58.12	8.27	19.61	3.7	58.5187	3	56.82	0.85
11.31	58	7	22	4.4	58	3	106	1.62
13.05	46.95	7	19	3.13	47	3	86	1.59
9.48	65.36	7	27	4.28	51	3	151	2.09
23.79	54.25	17	25	1.7	49	3	60	0.84
16.97	63.02	13	26	2.72	41	3	153	2.02

Perc_PTN	ADQ_PTN	GKG_LIP	PERCENTU	ADQ_LIP	MÉDIA_FIN	EAR_VITA	MÉDIA_FIN	EAR_B1
12.5802	3	0.319142	16.34937	3	230.4	2	0.64	2
16.12389	3	0.203598	10.90911	3	78	2	0.445	2
24.54568	3	0.389709	27.73868	1	298.5	2	0.32	2
10.95465	3	0.985117	26.55957	1	1026.3	1	0.81	2
32.01936	2	0.62	20.74	3	257.25	2	0.87	2
17.34525	3	0.82	33.33	1	74.9333	2	0.1933	2
16.00143	1	1.25	35.96	2	330.8	2	1.4567	1
6.947173	3	0.168507	12.04352	3	77.2	2	0.31	2
17.36293	1	0.955464	26.63727	1	253.65	1	1.29	1
14.1543	3	0.733452	22.48177	2	279.9	2	1.53	1
24.66199	1	0.514085	16.00526	3	89	2	0.595	2
18	2	1.64	30.31	1	533.57	3	1.6	1
34.98989	2	0.51928	19.50655	3	101.1667	1	0.59	2
62.09349	2	1.18	26.43	1	745.4	2	1.81	1
14	1	1.73	30.7	1	479.3	3	2.1	1
16.55584	1	0.73	20.92	3	11802.3	1	1.3	1
10.37111	3	0.95	18.86	3	269.7	2	1.19	1
19	3	0.71	26.15	1	723.5	3	1	1
29.96007	1	0.30549	12.14395	3	224.8	2	0.77	2
12.6197	1	1.53522	33.91534	2	409.95	2	0.88	2
15.90174	2	1.3	17.37	3	13670.96	1	2.23	1
18.82063	1	1	23.3	3	7481.767	1	1.576667	1
15.56216	1	0.77	17.07	3	7332.9	1	2.56	1
20.1078	1	0.9	34.14	1	167.8	2	0.89	2
18.29829	1	0.75	23.19	3	5639.66	1	1.07	1
21.98476	1	1.07	27.42	1	221.7	2	0.76	2
15.71744	3	0.75	24.77	3	281.7	2	0.52	2
29	2	0.62	19.27	3	197.43	3	1.5	1
19	1	1.2	29.71	1	239.34	3	1.71	1
19	1	1.23	32.4	1	3908.04	2	1.91	1
28.57143	1	0.536808	21.82289	1	247.3667	2	0.613333	2
28.95033	1	0.476796	20.49828	1	141.7333	2	0.603333	2
38.27139	1	1.06	32.39	1	1207.667	1	0.9067	2
26.19036	1	0.73	26.54	1	422.7667	2	0.7767	2
60.15031	1	0.77	26.6	1	266.4667	2	0.8267	2
17.16697	1	1.06	30.65	1	226.46	2	0.95	2
19.12528	1	1.06	27.91	1	4559.63	1	1.09	1
14.32979	3	0.33	12.88	3	13709.23	1	1.46	1
4.519234	3	0.03	9.88	3	3571.033	1	1.473333	1
24.3045	1	0.327053	11.6234	3	150.3667	2	1.09	1
17.50934	3	0.461848	32.13344	1	681.15	1	1.15	1
15.17913	1	0.947954	24.06648	1	402.2	2	1.09	1
15.02398	3	0.574381	18.47513	1	80.2	2	1.43	1
15.88026	1	1.05	27.47	1	881.4	1	1.646667	1
14.42119	1	1.35	33.09	1	3809.69	1	1.08	1
24.0474	1	0.8	22.96	3	267.63	2	1.21	1

19.82913	3	0.546074	25.76621	1	340.85	2	0.405	2
19.98029	1	1.17	35.07	2	628.27	2	1.77	1
15	1	1.01	29.28	1	225.27	3	1.13	1
25	2	1.24	33	1	595.32	3	1.82	1
20	1	0.85	27.08	1	251.51	3	0.99	1
19.07319	1	0.859447	27.43171	2	441.6	2	0.945	2
16.31399	3	0.65	25.4	1	119.4	2	0.97	2
8.969484	3	0.43	16.11	3	128.9	2	1.14	1
16.54813	3	0.41	24.53	3	160.6	2	0.883333	2
11.65702	1	1.580253	30.86425	2	454.8	2	1.455	1
52.05413	2	1.18	24.49	3	849.4	2	1.15	2
18	1	1.04	24.14	3	181.57	3	1.06	1
18	3	0.75	27.77	1	332.44	3	1.03	1
16	1	1.41	36.56	2	1015.64	1	2.33	1
27.94366	1	0.53	18.1	3	373	2	0.7933	2
18	3	0.51	20.46	3	186.86	3	1.12	1
20.14665	3	0.251549	14.13092	3	79.2	2	0.475	2
25.67236	3	0.21831	15.73378	3	66.2	2	0.46	2
25.57294	1	1.01	32.18	1	45.13333	2	0.926667	2
12	3	0.67	19.64	3	232.7	3	1.33	1
18.42151	3	0.480471	20.92608	1	464.5	2	0.66	2
13.53069	3	0.53	19.01	3	3443.3	1	1.16	1
21	1	0.7	20.6	3	261.26	3	1.14	1
24	1	0.88	29.52	1	767.53	1	0.84	3
25	2	0.9	24.21	3	2772.33	1	1.68	1
24	3	0.43	27.47	1	683.36	1	0.44	3
30	1	0.81	27.56	1	530.62	3	2.11	1

VITB2_FIN/	MÉDIA_FIN	EAR_VITB2	MÉDIA_FIN	EAR_VITB6	MÉDIA_FIN	EAR_B12	MÉDIA_FIN	EAR_VITC
0.88	0.88	2	0.25	2	1.21	2	19.95	2
1.44	0.72	2	0.13	2	0	2	27.75	2
2.48	0.826667	2	0.5	2	2.496667	1	13.56667	2
2.3	2.3	1	0.97	2	3.63	1	0	2
1.92	0.96	2	0.215	2	0.75	2	8.1	2
1.26	0.42	2	0.35	2	0.4033	2	5.8333	2
2.67	0.89	2	0.66	2	0	2	10.9667	2
1.05	0.525	2	0.315	2	2.735	1	0	2
3.27	1.635	1	0.35	2	3.645	1	26.65	2
2.4	1.2	1	0.54	2	1.825	2	4.2	2
1.8	0.9	2	0.49	2	4.295	1	5.2	2
10	3.32	1	2.86	1	4.92	1	97.82	1
1.88	0.626667	2	0.406667	2	3.29	1	44.5	2
6.75	2.25	2	1.1267	1	2.0233	1	77.9667	1
11	3.51	1	2.57	1	5.08	1	1378.86	1
14.95	4.98	1	2.46	1	122.82	1	79.6	1
2.58	0.86	2	1.303333	1	1.423333	2	36.46667	2
4	1.42	1	1.46	1	3.15	1	27.6	3
0.77	0.385	2	0.16	2	1.015	2	17.75	2
1.71	0.855	2	0.505	2	0.305	2	20.55	2
19.38	6.46	1	2.82	1	143.59	1	49.53	2
10.87	3.623333	1	2.053333	1	71.95667	1	69.53333	1
11.06	3.68	1	1.82	1	71.63	1	69.99	1
3.32	1.1	1	1.08	1	3.28	1	28.93	2
8.05	2.68	1	1.65	1	58.22	1	33.86	2
2.32	0.77	2	1.23	1	0.98	2	6.66	2
1.18	0.39	2	0.73	2	0.84	2	26.33	2
8	2.69	1	2.52	1	10.49	1	26.56	3
6	1.97	1	1.36	1	4.51	1	89.75	1
9	3	1	1.79	1	26.15	1	51.05	3
3.62	1.206667	1	0.523333	2	0.523333	2	0	2
2.88	0.96	2	0.326667	2	3.103333	1	0.6	2
2.25	0.75	2	0.2733	2	0.6	2	16.2	2
3.23	1.0767	2	0.2433	2	1.21	2	10.8	2
3.38	1.1267	2	0.2033	2	1.21	2	5.4	2
1.71	0.57	2	0.69	2	0.99	2	36.46	2
7.71	2.57	1	1.1	1	47.69	1	33.63	2
18.48	6.16	1	3.12	1	144.71	1	52.96	2
6.34	2.113333	1	2.646667	1	36.99667	1	62.96667	2
2.16	0.72	2	0.17	2	1.62	2	1	2
1.71	0.855	2	0.77	2	0.605	2	61.45	2
3.17	1.585	1	0.565	2	1.515	2	65.45	1
2.5	1.25	1	0.6	2	0	2	4.2	2
2.96	0.986667	2	0.86	2	1.1	2	33.85	2
5.64	1.88	1	1.45	1	36.73	1	31.99	2
4.21	1.4	1	1.82	1	2.51	1	19.83	2

2.64	1.32	1	0.345	2	3.03	1	1.5	2
6.2	2.06	1	1.99	1	7.18	1	48.86	2
5	1.54	1	1.42	1	0.59	3	24.06	3
6	2.02	1	2.25	1	6.24	1	104.41	1
3	1.07	1	1.45	1	3.51	1	128.94	1
2.1	1.05	2	0.455	2	2.425	1	96.95	1
1.72	0.57	2	0.55	2	1.05	2	27.73	2
2.4	0.8	2	0.89	2	1.45	2	24.63	2
1.57	0.523333	2	0.82	2	0.333333	2	29.76667	2
1.62	0.81	2	0.285	2	0.605	2	95.1	1
3.48	1.16	2	0.5733	2	0.61	2	49.7	2
3	1.14	1	1.61	1	3.51	1	206.96	1
5	1.6	1	1.6	1	4.25	1	203.57	1
6	2.16	1	1.62	1	1.8	3	77.98	1
1.52	0.5067	2	0.1967	2	0	#NULL!	30.4	2
5	1.71	1	1.73	1	2.8	1	3945.92	2
0.95	0.475	2	0.125	2	1.215	2	60.45	2
1.23	0.615	2	0.295	2	2.735	1	0	2
4.07	1.356667	1	1.616667	1	7.066667	1	10.3	2
7	2.24	1	1.69	1	1.49	3	80.08	1
1.71	0.855	2	0.35	2	0.605	2	5.1	2
5.04	1.68	1	0.88	2	36.87	1	45.69	2
4	1.34	1	1.63	1	6.14	1	92.49	1
3	1.09	1	1.13	1	6.58	1	99.33	1
7	2.24	1	2.58	1	9.82	1	207.38	1
2	0.65	3	1.32	1	2.16	1	67.31	1
5	1.82	1	1.83	1	5.27	1	116.73	1

MÉDIA_FIN	EAR_VITE	MÉDIA_FIN	EAR_FOL	MÉDIA_FIN	EAR_CA	MÉDIA_FIN	EAR_P	EAR_MG
4.9	2	50.5	2	204.4	2	592.9	2	2
0	2	3.3	2	244	2	707.6	2	2
1.4	2	45.73333	2	176.5667	2	615.4	2	2
4.9	2	143.5	2	401.5	2	901	2	2
0.65	2	33.5	2	394.15	2	1700.05	1	2
0.3667	2	15.9333	2	85	2	635.4667	2	2
0.0333	2	3.3333	2	187.2667	2	943.8	2	2
0.5	2	20.05	2	122.45	2	496.6	2	2
1.15	2	43.35	2	479.1	2	1231.05	1	2
0.85	2	28.65	2	291.75	2	962.95	2	2
0.15	2	21.25	2	172.2	2	1045.6	1	2
5.22	3	263.5	2	616.21	2	1433.14	1	2
3.7	2	18.16667	2	284.1667	2	979.6	2	2
1.5667	2	129.52	2	944.1	2	1921.967	1	2
5.19	3	385.1	1	779.74	2	1590.12	1	2
4.6	2	286.03	2	285.73	2	1140.7	1	2
9.866667	2	55.5	2	307.3333	2	1086.233	2	2
2.34	3	161.39	2	172.66	2	820.26	2	2
0.05	2	3.4	2	139.6	2	1122.3	1	2
0.4	2	13.6	2	162.65	2	891.5	2	2
6.5	2	310.23	2	418.2	2	1985	1	2
17.43333	1	210.9667	2	266.8667	2	1280	1	2
17.41	1	218.25	2	428.41	2	1408.12	1	2
13.76	1	53.73	2	288.4	2	959.66	2	2
8.56	2	148.46	2	222.23	2	1008.96	2	2
25.13	1	47.83	2	158.8	2	1129.3	1	2
4.6	2	30.3	2	208.86	2	476.96	2	2
4.67	3	163.17	2	393.05	2	1663.44	1	2
2.25	2	198.89	2	366.13	2	1167.06	1	2
3.52	3	364.77	1	543.95	2	1660.47	1	2
1.3	2	56.4	2	131.9667	2	925.3333	2	2
1.233333	2	42.83333	2	155.4333	2	861.6	2	2
0.4667	2	24.0667	2	459.1667	2	1618.933	1	2
1.1333	2	55.1333	2	267.9	2	1419.7	1	2
1.1333	2	54.8	2	302.1	2	1547.767	1	2
5.96	2	36.86	2	151.5	2	822.53	2	2
7.6	2	133	2	537	2	1239.06	1	2
14.8	1	324.36	2	266.9	2	1604.3	1	2
7.533333	2	129.3333	2	176.8667	2	1236.633	1	2
0.1	2	8.066667	2	169.8	2	1032.5	1	2
0.8	2	23.9	2	530.85	2	889.8	2	2
2.25	2	68.15	2	376.2	2	1136.45	1	2
0	2	4	2	211.05	2	984.6	2	2
10.26667	2	82.63333	2	319.5	2	982.0667	2	2
4.54	2	94.28	2	234.86	2	942.06	1	2
11.03	2	71.43	2	507.03	2	1400.16	1	2

2.5	2	78.15	2	254.15	2	808.2	2	2
23.77	1	144.36	2	439.77	2	1359.68	1	2
2.34	3	131.19	2	251.67	2	744.24	2	2
1.86	3	65.67	2	839.99	2	1511.13	1	2
2.97	3	292.11	2	185.97	2	815.77	2	2
1.65	2	53.25	2	226.15	2	1210.35	1	2
4.3	2	36.3	2	298.8	2	405.9	2	2
10.7	2	52.33	2	450.3	2	910.2	2	2
6.233333	2	68.8	2	161.5	2	493.3667	2	2
0.8	2	23.9	2	255	2	1145.35	1	2
0.7	2	33.9	2	1000.967	2	2380.267	1	2
3.53	3	150.98	2	215.92	2	980.06	2	2
4.24	3	241.15	2	232.96	2	804.02	2	2
2.64	3	192.17	2	504.19	2	1289.1	1	2
0.0333	2	1.8	2	195.4333	2	924.1333	2	2
2.5	3	161.36	2	203.51	2	823.96	2	2
0.05	2	5.7	2	195.2	2	713.05	2	2
0.5	2	20.05	2	124.1	2	602.35	2	2
5.933333	2	71.03333	2	157.7	2	991.8333	2	2
2.49	3	147.71	2	227.11	2	827.58	2	2
0.8	2	26.25	2	255.4	2	881	2	2
3.8	2	101.16	2	174.86	2	744.4	2	2
3.53	3	171.53	2	325.45	2	1249.02	1	2
4.65	3	232.5	2	178.29	2	903.78	2	2
7.66	3	292.9	2	1025.23	2	1950.6	1	2
2.38	3	63.3	2	132.03	2	665.55	2	2
2.37	3	107.7	2	161.54	2	1417.29	1	2

MÉDIA_FIN	EAR_FE	MÉDIA_FIN	EAR_ZN	MÉDIA_T	EAR_SE	MÉDIA_PO	EAR_K	MÉDIA_FIN
9.3	1	1.3	2	36.3	2	397.6	2	156.7
7.05	1	2.9	2	0	#NULL!	797.25	2	197.2
6.366667	2	5.133333	2	48.4	1	504.0333	2	449.5333
7.9	1	4.8	2	108.8	1	397.6	2	670.7
19.15	1	23.65	1	25.45	2	1584.1	2	600.4
5.7667	2	9.0667	1	12.1	2	1090.333	2	655.9
9.9333	1	8.4333	2	2.4667	2	1095.167	2	1993.067
9.55	1	4.5	2	38.75	2	306.65	2	210.6
14.45	1	7.8	2	55.25	1	1304.45	2	986.85
12.8	1	4.3	2	33	2	599.3	2	985.75
11.15	1	7.8	2	66.85	1	1041.3	2	794.05
15.67	1	14.2	1	115.19	1	3074.27	2	2150
9.633333	1	5.566667	2	63.4	1	1146.067	2	900.9333
14.8667	1	17.2333	1	74.8	1	2174.7	2	1765.767
19	1	15.94	1	126.75	1	3348.66	2	4939
17.03	1	10.63	1	121.1	1	1581.7	2	2219.8
16.66667	1	9.366667	2	67.26667	1	2607.6	2	3199.967
10.26	1	9.73	1	79.78	1	1730.18	2	2087
6.3	2	4.3	2	12.25	2	1083.4	2	190.2
9.75	1	3.7	2	9.05	2	777.2	2	1469.45
26.26	1	12.6	2	129.8	1	1711	1	3508.43
18.3	1	9.833333	1	108	1	1852.767	2	2481.833
28.57	1	8.57	2	108.44	1	1934.72	1	3539.27
11.3	1	9.63	2	74.16	1	1329	1	1659.73
13.93	1	8.23	2	101	1	1387.4	2	2255.66
13.83	1	10.6	1	72.56	1	1632.23	2	3440.4
6.4	2	4.8	2	27.46	2	729.83	2	1514.7
8.77	1	8.97	1	237.34	1	2488.28	2	2188
11.11	1	13.08	1	94.26	1	2258.92	2	3170
14.69	1	12.96	1	76.41	1	2415.24	2	3245
11.2	1	8.133333	2	74.8	1	650.6333	2	408.1667
11.03333	1	6.6	2	52.23333	1	586.4333	2	198.7667
12.8667	1	14	1	17.1667	2	1057.733	2	713.1333
16.7667	1	19.5	1	39.3	2	1260	2	340.6667
16.1	1	17.9333	1	40.2667	2	1466.1	2	401.0667
11.46	1	8.26	2	56.53	1	1276.73	2	1543.26
12.66	1	8.13	2	61.76	1	1834.66	1	2093.8
21.46	1	13.83	1	147.8	1	1513.133	2	2770.567
15.66667	1	7.133333	2	135.5667	1	1434.033	2	2870.967
14.36667	1	4.666667	2	19.83333	2	488.0333	2	97.6
14.55	1	6.45	2	18.15	2	615.35	2	571.2
15.15	1	5.5	2	45.35	1	1016.6	2	848.3
9.45	1	3.35	2	0.05	2	1017.2	2	578.85
15.73333	1	5.833333	2	59.6	1	1888.033	2	1876.233
12.74	1	4.86	2	67.9	1	1051.03	1	2298.24
12.03	1	6.86	2	149.93	1	1685.43	1	1738.86

7.95	1	4.45	2	69.3	1	428.95	2	361.75	
20.66	1	18.43	2	105.9	1	2369.89	1	2476.35	
9.66	1	7.25	2	45.8	1	1956	2	2500	
13.85	1	19.75	1	140.07	1	1949.29	2	1948	
11.58	1	11.66	1	114.9	1	1886.07	2	713	
11.6	1	7.25	2	51.15	1	1282.75	2	648.9	
6.63	2	5.46	2	24	2	1086.73	2	912.26	
11.63	1	5.7	2	58.8	1	1559.8	1	1807.66	
10.8	1	4.5	2	30.03333	2	501	2	909.7	
12.25	1	4.6	2	18.15	2	823	2	1587.15	
15.9667	1	15.7333	1	25.5	2	3071.133	2	1108.967	
10.86	1	11.02	1	113.67	1	1531.87	2	1799	
10.86	1	10.55	1	76.19	1	2275.33	2	1166	
10.39	1	9.8	1	60.93	1	2283.11	2	3843	
12.7333	1	10.8667	1	0.2333	2	1383.033	2	387.3667	
10.54	1	9.94	1	64.5	1	1927.37	2	1764	
8	1	3.1	2	14.85	2	447.7	2	235.25	
7.65	1	5.4	2	38.75	2	38.75	2	172.45	
14.26667	1	15.73333	1	76.13333	1	1465.733	2	1760.367	
6.95	2	5.35	2	51.78	1	1100.06	2	3025	
7.9	1	3.6	2	18.15	2	659.3	2	240.8	
12.73	1	6.53	2	63.83	1	1027.6	2	2222.03	
9.87	1	9.18	1	150.41	1	2339.76	2	1993	
12	1	14.78	1	102.49	1	2300.88	2	845	
17.44	1	13.9	1	227.68	1	3993.04	2	2974	
5.16	2	4.71	2	70.49	1	1549	2	340	
12.76	1	21.67	1	139.67	1	2767.77	2	1023	

EAR_NA	Sat_percen	@_GSAT	Poli_percei	@_GPOLI	Mono_Perc	@_GMON	MÉDIA_Fib	Reco_Fibra
1	0	1	0.470711	3	0	3	6.6	22
1	2.875118	1	1.078169	3	2.417712	3	4.05	22
1	3.241258	1	2.13923	3	2.722656	3	4.7	21
1	1.397635	1	0.27051	3	0.766445	3	6.5	20
2	7.799702	1	2.366962	3	7.033258	3	6.5	20
2	9.767263	1	2.89967	3	9.404768	3	6.566667	21
1	6.219472	1	2.487796	3	6.642383	3	9.2	23
1	0.030855	1	0.046282	3	0.030855	3	8.3	22
1	5.04638	1	2.418454	3	4.170405	3	11.25	23
1	1.132034	1	0.674867	3	1.153804	3	10.4	22
1	2.768033	1	1.891033	3	2.576189	3	6.5	20
1	10	1	6	1	10	3	23.69	19
1	4.413152	1	2.8506	3	3.969725	3	4.5	24
1	24.7873	2	4.987872	3	19.4703	1	4.233333	19
2	11	2	5	3	11	1	27.4	21
1	16.03453	2	2.597142	3	5.744765	3	14.03333	24
2	6.289366	1	3.611715	3	5.905362	3	21.56667	21
1	9	1	5	3	9	3	23.29	22
1	1.978885	1	1.338658	3	2.153493	3	2.95	24
1	6.325191	1	6.082632	1	7.220793	3	9.2	23
1	4.864134	1	2.825503	3	3.968661	3	15.93	25
2	5.675846	1	6.072133	1	7.107591	3	12.2	22
1	8.943191	1	4.194798	3	5.947848	3	16.57	22
1	10.30001	2	6.711991	1	12.45282	1	4.26	22
1	7.576301	1	4.445598	3	7.899004	3	13.06	23
2	8.085144	1	7.506453	1	9.077186	3	17.96	23
1	8.397093	1	2.229768	3	5.276551	3	6.8	21
1	6	1	5	3	6	3	14.48	23
2	11	2	4	3	11	1	27.06	22
2	11	2	6	1	12	1	26.21	23
1	1.147644	1	1.235924	3	1.368345	3	4.633333	20
1	1.822969	1	1.392546	3	2.253393	3	4.233333	19
2	17.32497	2	2.696697	3	14.99687	1	2.95	24
2	7.308245	1	3.18748	3	7.566689	3	14.05	21
2	15.74919	2	9.658696	1	17.31796	1	1.6	21
1	11.55369	2	4.330096	3	11.11713	1	8.26	21
1	8.854674	1	3.391249	3	5.915287	3	16.66	21
2	8.079405	1	5.543763	3	8.520386	3	5.933333	22
2	6.693007	1	3.141327	3	6.353404	3	8.4	24
1	1.449825	1	1.163676	3	1.774128	3	6.566667	21
1	8.573313	1	2.555507	3	6.059024	3	2.8	23
1	4.487535	1	3.157895	3	4.342105	3	12.15	22
1	5.126778	1	1.849386	3	5.173598	3	10.4	23
1	10.752	2	4.116882	3	8.941133	3	14.1	23
1	11.61647	2	1.777597	3	7.165938	3	5.18	24
1	8.326036	1	4.456483	3	6.565243	3	11.76	23

1	1.43301	1	0.209709	3	0.524272	3	5.1	21
1	11.07521	2	6.896484	1	12.677	1	12.84	21
2	11	2	5	3	11	1	18.17	21
1	14	2	3	3	12	1	25.02	23
1	8	1	6	1	10	3	11.68	22
1	3.919739	1	4.824294	3	4.867368	3	14.05	21
2	10.02621	2	3.346962	3	7.530664	3	7.36	21
1	5.880583	1	4.656816	3	5.284944	3	17.36	21
2	10.17126	2	2.611204	3	7.33624	3	5.733333	24
1	2.005574	1	1.203345	3	2.098139	3	8.3	22
2	11.09039	2	4.110941	3	10.34132	1	6.5	20
1	7	1	5	3	8	3	16.76	21
1	11	2	3	3	10	3	17.48	23
2	13	2	6	1	12	1	20.41	22
2	4.855394	1	1.809724	3	6.267923	3	4.633333	20
1	6	1	5	3	7	3	21.16	20
1	3.852757	1	0.579985	3	2.31994	3	2.85	23
1	2.076306	1	0.876662	3	1.845605	3	1.6	21
1	11.0043	2	2.407191	3	12.21695	1	10.83333	22
2	7	1	4	3	7	3	18.08	22
1	3.508088	1	3.938325	3	3.640469	3	8.05	22
1	6.911798	1	2.5504	3	6.622467	3	14	24
1	7	1	5	3	7	3	23.75	23
1	10	1	4	3	12	1	16.26	19
2	10	1	5	3	8	3	26.62	23
1	8	1	7	1	9	3	18	22
1	10	1	3	3	11	1	17.32	20

ADQ_FIB	MÉDIA_AC	EAR_B3	MEDIA_KC/	KCALKG	Time	divisao	Carga_trei	NAF
2	3.92	1	1147.2	17.56815	3	1	15	2
2	15.805	2	1377.335	16.79677	3	2	15	2
2	6.023333	1	925.5667	12.64435	3	3	15	2
2	2.53	1	1996.23	33.38177	3	1	15	2
2	2.53	1	1996.23	33.38177	2	2	10	2
2	15.21	2	1572.603	25.32373	2	3	10	2
2	14.845	2	2411.785	40.73961	2	3	15	2
2	17.81	2	2916.87	46.0801	3	2	15	2
2	9.63	1	2363.08	32.28251	3	2	15	2
2	16.58	2	2067.075	29.36186	3	2	15	2
2	6.135	1	1641.96	28.90775	3	2	15	2
1	24.24	1	2670	49	4	3	6	1.8
2	17.04667	2	1420.753	23.95874	3	3	15	2
2	11.48	1	1184.88	17.6	2	3	10	2
1	29.31	1	3557	51	4	3	6	1.8
2	33.50667	1	2125.413	31.81	1	3	15	2
2	18.87667	1	2890.593	45.66	1	3	15	2
1	17.81	1	1642	24	4	2	6	1.8
2	22.455	2	1546.325	22.64019	3	2	15	2
2	14.845	2	2411.785	40.73961	3	2	15	2
2	45.6	1	4723.76	67.48	1	3	15	2
2	30.04333	1	2346.787	38.78	1	3	15	2
2	29.53	1	2731.24	40.64	1	3	15	2
2	13.72	1	1881.26	29.21	1	3	15	2
2	28.86	1	1868.59	29.15	1	3	15	2
2	23.84	1	2177.327	35.23	1	3	15	2
2	12.222	1	1586.263	27.49	1	3	15	2
3	15.33	1	1866	29	4	3	6	1.8
1	21.26	1	2000	36	4	3	6	1.8
1	28.15	1	2112	34	4	3	6	1.8
2	6.763333	1	1359.307	22.13855	3	3	15	2
2	11.48	1	1184.88	20.93428	3	3	15	2
2	22.455	2	1546.325	22.64019	2	3	10	2
2	15.5	2	2089.425	28.19737	2	3	10	2
2	5.375	1	975.29	12.48771	2	3	10	2
2	14.59	1	1772.94	31.32	1	3	15	2
2	31.93	1	1971.84	34.3	1	3	15	2
2	43.97667	1	1904.843	23.2	1	3	15	2
2	51.78667	1	2120.123	28.15	1	3	15	2
2	15.21	2	1572.603	25.32373	3	3	15	2
2	14.635	2	1091.76	12.93555	3	2	15	2
2	18.68	2	2166	35.45008	1	2	15	2
2	12.15	2	1922.26	27.9805	3	2	15	2
2	20.47333	1	2120.537	34.79	1	3	15	2
2	28.53	1	2754.28	36.92	1	3	15	2
2	42.03	1	2146.76	31.38	1	3	15	2

2	3.385	1	1287.5	19.07407	3	2	15	2
2	32.05	1	2556.52	30.25	1	3	15	2
3	16.19	1	1788	31	4	3	6	1.8
1	28.4	1	2326	34	4	3	6	1.8
3	23.92	1	1696	28	4	3	6	1.8
2	15.5	2	2089.425	28.19737	3	2	15	2
2	6.66	2	1226.187	23.22	1	3	15	2
2	16.43	1	1662.08	24.22	1	3	15	2
2	11.55333	2	1206.34	15.09	1	3	15	2
2	17.81	2	2916.87	46.0801	3	2	15	2
2	6.135	1	1641.96	24.41	2	3	10	2
3	19.3	1	2188	39	4	3	6	1.8
3	17.95	1	1566	24	4	3	6	1.8
3	28.52	1	2228	35	4	3	6	1.8
2	6.763333	1	1359.307	22.13855	2	3	10	2
1	16.89	1	1686	22	4	3	6	1.8
2	6.79	1	1086.235	16.02117	3	2	15	2
2	5.375	1	975.29	12.48771	3	2	15	2
2	22.55	1	1657.533	28.43	1	3	15	2
3	11.42	2	1853	31	4	3	6	1.8
2	17.095	2	1359.715	20.66436	3	2	15	2
2	15.84	1	1679.737	25.29	1	3	15	2
1	22	1	1987	30	4	3	6	1.8
3	15.37	1	1442	27	4	3	6	1.8
1	25.19	1	2427	34	4	3	6	1.8
3	11.37	2	994	14	4	3	6	1.8
3	37.48	1	2018	27	4	3	6	1.8

NAF_IPAQ	novo_sub_	TMB	Goldberg_2	EI_TMB	Sub_relato	IDADE	ALEM_FUT	QUAL
#NULL!	1	1812.896	1.12	0.6328	1	17	1	1
#NULL!	1	2108.252	1.24	0.653307	1	17	2	9
#NULL!	1	1952.615	1.3	0.474014	1	16	2	9
#NULL!	1	1715.623	1.12	1.16356	2	15	1	4
#NULL!	1	2037.508	1.24	0.979741	1	17	2	9
#NULL!	1	1791.673	1.3	0.877729	1	19	1	
#NULL!	2	1765.144	1.3	1.366339	2	16	2	9
#NULL!	2	1842.962	1.24	1.582708	2	17	1	4
#NULL!	1	1952.615	1.24	1.210213	1	18	1	1
#NULL!	1	1903.094	1.24	1.086165	1	17	1	2
#NULL!	1	1662.565	1.24	0.987607	1	15	2	9
#NULL!	2	1628.961	1.16	1.639081	2	14	1	1
#NULL!	1	1706.78	1.3	0.832417	1	19	2	9
#NULL!	1	1846.499	1.3	0.64169	1	16	2	9
#NULL!	2	1901.326	1.16	1.8708	2	16	1	1
#NULL!	1	1839.425	1.3	1.155477	1	19	2	9
#NULL!	2	1777.524	1.3	1.626191	2	16	2	9
#NULL!	1	1850.036	1.12	0.88755	1	17	2	9
#NULL!	1	1865.954	1.24	0.828705	1	19	2	9
#NULL!	2	1705.011	1.24	1.414527	2	18	2	9
#NULL!	2	1896.02	1.3	2.491408	2	20	2	9
#NULL!	2	1728.003	1.3	1.358092	2	17	2	9
#NULL!	2	1846.499	1.3	1.479145	2	17	2	9
#NULL!	1	1796.978	1.3	1.046902	1	17	2	9
#NULL!	1	1791.673	1.3	1.04293	1	18	2	9
#NULL!	2	1750.995	1.3	1.24348	1	18	2	9
#NULL!	1	1678.482	1.3	0.945058	1	16	1	1
#NULL!	1	1793.441	1.16	1.040458	2	19	1	1
#NULL!	2	1627.193	1.16	1.229111	2	17	2	9
#NULL!	2	1747.458	1.16	1.208613	2	18	1	2
#NULL!	1	1743.92	1.3	0.779455	1	15	2	9
#NULL!	1	1659.028	1.3	0.714201	1	14	2	9
#NULL!	1	2016.285	1.3	0.766918	1	19	2	9
#NULL!	1	1920.78	1.3	1.0878	1	18	2	9
#NULL!	1	1966.764	1.3	0.495886	1	17	2	9
#NULL!	1	1659.028	1.3	1.068662	1	16	1	2
#NULL!	1	1673.176	1.3	1.178501	1	16	2	9
#NULL!	1	2110.021	1.3	0.902761	1	17	1	1
#NULL!	1	1989.756	1.3	1.065519	1	19	1	1
#NULL!	1	1756.301	1.3	0.895407	1	16	2	9
#NULL!	1	2150.698	1.24	0.50763	1	18	2	9
#NULL!	1	1738.615	1.24	1.24582	2	17	1	4
#NULL!	1	1873.028	1.24	1.026285	1	18	1	2
#NULL!	1	1738.615	1.3	1.21967	1	18	1	1
#NULL!	2	1977.376	1.3	1.392897	2	19	2	9
#NULL!	1	1867.722	1.3	1.1494	1	18	2	9

#NULL!	1	1851.805	1.24	0.695268	1	16	2	9
#NULL!	1	2152.467	1.3	1.187716	1	16	2	9
#NULL!	1	1674.945	1.16	1.067498	1	16	1	2
#NULL!	2	1878.334	1.16	1.238331	2	18	2	9
#NULL!	1	1722.697	1.16	0.984503	1	17	1	2
#NULL!	1	1968.533	1.24	1.061412	1	16	2	9
#NULL!	1	1591.821	1.3	0.770304	1	16	2	9
#NULL!	1	1871.26	1.3	0.888215	1	16	2	9
#NULL!	1	2071.111	1.3	0.58246	1	19	2	9
#NULL!	2	1777.524	1.24	1.640974	2	17	2	9
#NULL!	1	1846.499	1.3	0.889229	1	19	2	9
#NULL!	2	1659.028	1.16	1.318845	2	16	2	9
#NULL!	1	1798.747	1.16	0.870606	1	18	2	9
#NULL!	2	1789.904	1.16	1.244759	2	17	1	1
#NULL!	1	1765.144	1.3	0.770083	1	17	2	9
#NULL!	1	1987.987	1.16	0.848094	1	15	2	9
#NULL!	1	1857.111	1.24	0.584906	1	18	1	1
#NULL!	1	2039.277	1.24	0.478253	1	16	1	4
#NULL!	1	1689.094	1.3	0.981315	1	17	1	3
#NULL!	1	1729.772	1.16	1.07124	1	17	1	1
#NULL!	1	1821.739	1.24	0.746383	1	17	2	9
#NULL!	1	1832.35	1.3	0.916711	1	19	2	9
#NULL!	1	1814.664	1.16	1.094968	1	18	1	1
#NULL!	1	1613.044	1.16	0.893962	1	14	2	9
#NULL!	2	1934.929	1.16	1.254309	2	18	1	1
#NULL!	1	1917.243	1.16	0.518453	1	17	1	1
#NULL!	1	2000.367	1.16	1.008815	1	15	1	2

POS_JOGO	Pos_jogo_2	Pos_jogo_3	Posição_go	TEMPO	FOI_NUTRI	INFO_NUT	INF_NUT_C	ÁGUA_TRE
8	8	5	2	7	2	5	2	3
2	2	2	2	5	2	4	2	1
8	8	5	2	3	2	1	2	0.6
3	3	3	2	2	2	1	2	0.5
1	1	1	1	10	1	2	2	1.5
8	8	5	2	9	2	5	2	1
3	3	3	2	4	2	5	2	1
1	1	1	1	10	2	1	2	1
4	4	4	2	6	2	4	2	0.3
5	5	5	2	9	2	4	2	1
2	2	2	2	7	2	1	2	1
8	8	5	2	6	2	7	0	1
3	3	3	2	12	2	4	2	0.5
5	5	5	2	6	1	8	1	1
8	8	5	2	6	1	5	0	1
5	5	5	2	10	2	5	2	0.5
8	8	5	2	6	2	5	2	2
3	3	3	2	7	2	3	0	2
4	4	4	2	9	1	8	1	1
5	5	5	2	6	2	2	2	1
3	3	3	2	4	2	1	2	1
2	2	2	2	5	2	1	2	0.5
3	3	3	2	13	2	4	2	2
7	5	5	2	8	2	2	2	2
8	8	5	2	6	2	5	2	2
6	5	5	2	12	2	4	2	1.5
5	5	5	2	10	2	5	2	1
4	4	4	2	12	2	3	4	1
5	5	5	2	10	2	2	0	1
5	5	5	2	7	2	2	0	1
5	5	5	2	10	2	1	2	1
3	3	3	2	5	1	8	1	1
4	4	4	2	12	2	1	2	1.5
3	3	3	2	6	2	4	2	1
2	2	2	2	#NULL!	1	8	1	1
1	1	1	1	9	2	3	2	3
5	5	5	2	10	2	8	1	1
1	1	1	1	3	2	3	2	2
8	8	5	2	9	2	5	2	2
4	4	4	2	6	1	8	1	1.5
1	1	1	2	9	2	4	2	0.4
5	5	5	2	4	2	3	2	0.5
2	2	2	2	7	2	1	2	0.5
2	2	2	2	7	2	4	2	1
5	5	5	2	8	1	5	2	2
2	2	2	2	4	2	9	2	0.5

4	4	4	2	8	2	4	2	1
1	1	1	1	5	2	4	2	1.5
3	3	3	2	8	2	7	0	1
2	2	2	2	7	2	3	4	2
4	4	4	2	6	2	3	0	1
8	8	5	2	6	2	1	2	1
5	5	5	2	6	2	7	2	1
2	2	2	2	9	1	5	2	1
4	4	4	2	10	2	5	2	1
8	8	5	2	10	2	5	2	0.5
5	5	5	2	10	2	5	2	1
3	3	3	2	11	2	1	0	1
4	4	4	2	7	2	5	0	1
2	2	2	2	7	2	4	0	1
4	4	4	2	10	1	5	2	1
2	2	2	2	5	2	1	0	1
8	8	5	2	9	2	4	2	1
8	8	5	2	6	1	5	2	1.5
4	4	4	2	7	2	3	2	1
3	3	3	2	8	2	1	0	1
3	3	3	2	3	2	5	2	0.5
4	4	4	2	8	2	1	2	2
4	4	4	2	4	2	4	5	2
8	8	5	2	5	2	3	0	1
8	8	5	2	8	2	3	0	1
1	1	1	1	9	2	9	0	1
1	1	1	1	4	2	6	0	1

ÁGUA_DIA	REFEIÇÃO_	Adeq_refei	DIFICULDA	HORÁRIO_I	VIDA_CORI	FORÇA_VO	N_MUDAR_	HABILIDAD
7	2	2	#NULL!	2	2	2	2	2
6	3	1	#NULL!	2	2	2	2	2
2	3	1	#NULL!	2	2	2	2	2
3	3	1	#NULL!	2	2	2	2	2
2.5	2	2	#NULL!	2	2	2	2	2
2	2	2	#NULL!	2	2	2	2	2
2	2	2	#NULL!	2	2	2	2	2
2	2	2	#NULL!	2	2	2	2	2
3.5	3	1	#NULL!	2	2	2	2	2
2.5	3	1	#NULL!	2	2	1	2	2
1	3	1	#NULL!	2	2	1	2	2
3	1	2	#NULL!	2	2	2	2	2
2.5	1	2	#NULL!	2	1	2	2	2
2	3	1	#NULL!	2	2	2	2	2
2	3	1	#NULL!	2	2	2	2	2
1.5	2	2	#NULL!	2	2	2	1	2
5	2	2	#NULL!	2	1	2	2	2
3	1	2	#NULL!	2	2	2	2	2
2	1	2	#NULL!	2	2	1	2	2
2	3	1	#NULL!	2	2	2	2	2
2.5	2	2	#NULL!	2	2	2	2	2
1	2	2	#NULL!	2	2	2	2	2
3	1	2	#NULL!	2	1	2	2	2
5	3	1	#NULL!	2	2	2	1	2
6	1	2	#NULL!	2	2	2	2	2
2.5	2	2	#NULL!	2	1	2	2	2
2.5	3	1	#NULL!	2	2	2	2	2
3	1	2	#NULL!	2	2	2	2	2
2	1	2	#NULL!	2	1	2	2	2
2	2	2	#NULL!	2	2	2	2	1
2	2	2	#NULL!	2	2	1	2	2
1.2	2	2	#NULL!	2	2	1	2	2
2	2	2	#NULL!	2	2	2	2	2
2	1	2	#NULL!	2	2	2	2	2
2	2	2	#NULL!	2	2	2	1	2
4	2	2	#NULL!	2	2	2	2	2
2	1	2	#NULL!	2	2	2	2	2
3	2	2	#NULL!	2	2	2	2	2
1.5	1	2	#NULL!	2	2	2	2	2
2	2	2	#NULL!	2	2	2	2	2
2	3	1	#NULL!	2	2	1	2	2
5	2	2	#NULL!	2	2	2	2	2
1.5	2	2	#NULL!	2	2	1	2	2
2	3	1	#NULL!	2	1	2	2	2
2	2	2	#NULL!	2	2	1	2	1
1	2	2	#NULL!	2	2	2	1	2

2	3	1	#NULL!	2	1	2	2	2
2.5	3	1	#NULL!	2	2	2	2	1
2	1	2	#NULL!	2	1	2	2	2
3	2	2	#NULL!	2	2	2	2	1
4	3	1	#NULL!	2	2	2	1	2
2	2	2	#NULL!	2	2	1	2	2
2	2	2	#NULL!	2	2	1	2	2
1.5	2	2	#NULL!	2	1	2	2	2
3	2	2	#NULL!	2	2	2	2	2
2.5	2	2	#NULL!	2	2	1	2	2
2	3	1	#NULL!	2	1	2	2	1
3	2	2	#NULL!	2	2	2	2	1
3	1	2	#NULL!	2	2	2	2	1
2	3	1	#NULL!	2	2	2	2	2
2.5	3	1	#NULL!	2	1	2	2	2
4	2	2	#NULL!	2	2	2	2	1
2	2	2	#NULL!	1	2	1	2	2
2	2	2	#NULL!	1	2	2	2	2
1	3	1	#NULL!	2	1	2	2	2
2	2	2	#NULL!	2	2	2	2	1
1.5	2	2	#NULL!	2	1	1	2	2
5	2	2	#NULL!	2	2	1	2	2
3	3	1	#NULL!	2	2	1	2	2
2	2	2	#NULL!	2	1	2	2	1
5	3	1	#NULL!	2	2	2	1	2
3	2	2	#NULL!	2	2	1	2	2
2	2	2	#NULL!	1	1	2	2	1

COMIDA_S	PREP_LENT	DIFÍCIL_AR	PREÇO_CO	COMIDA_S	COMIDA_E	DESISTI_CC	CHAMAR_A	PREF_FAM
2	2	2	2	2	2	2	2	2
2	2	2	2	2	2	2	2	2
2	2	2	2	2	2	2	2	2
2	2	2	2	2	2	2	2	2
2	2	2	2	2	2	2	2	2
2	2	2	2	2	2	2	2	2
2	2	2	2	2	2	2	2	2
2	2	2	2	2	2	2	2	2
2	2	2	2	2	2	2	2	2
2	2	2	2	2	2	2	2	2
2	2	2	2	2	2	2	2	2
2	2	2	2	2	2	2	2	2
2	2	2	2	2	2	2	2	2
2	2	2	2	2	2	2	2	2
2	2	2	2	2	2	2	2	1
2	2	2	2	2	2	2	2	2
2	2	2	2	2	2	2	2	2
2	2	2	2	2	2	1	2	2
2	2	2	2	2	2	2	2	2
2	2	2	2	2	2	2	2	2
2	2	2	2	2	2	2	2	2
2	2	2	2	2	2	2	2	2
2	2	2	2	2	2	2	2	2
2	2	2	2	2	2	2	2	2
2	2	2	2	2	1	2	2	2
2	2	2	2	2	2	2	2	2
2	2	2	2	2	2	2	2	2
2	1	2	2	2	2	2	2	2
2	2	2	2	2	2	2	2	2
2	2	2	2	2	2	2	2	2
2	2	2	2	2	2	2	2	2
2	2	2	2	2	2	2	2	2
2	2	2	2	2	2	2	2	2
2	2	2	2	2	2	2	2	2
2	2	2	2	2	2	2	2	2
2	2	2	2	2	1	2	2	1
2	2	2	2	2	2	2	2	1
2	2	2	2	2	2	2	2	1
2	2	2	2	2	2	2	2	2
2	2	2	2	2	2	2	2	2
2	2	2	1	2	2	2	2	2
1	2	2	1	2	2	2	2	2
2	2	2	2	2	2	2	2	2
2	2	2	2	2	2	2	2	2
2	2	2	2	2	2	2	2	2
2	2	2	2	2	2	2	2	2

2	2	2	2	2	2	2	2	2
2	2	2	2	2	2	2	2	1
2	2	2	2	2	2	2	2	1
2	2	2	2	2	2	2	2	2
1	2	2	2	2	2	2	2	2
2	2	2	2	2	2	2	2	2
2	2	2	2	1	2	2	2	2
2	2	2	2	2	1	2	2	2
2	2	2	1	2	2	2	2	2
2	2	2	2	2	2	2	2	1
2	2	2	2	2	2	2	2	2
2	2	2	2	2	2	2	2	2
2	2	2	1	2	2	2	2	2
2	2	2	2	2	2	1	2	1
2	1	2	1	2	2	2	2	2
2	2	1	2	2	2	2	2	2
2	2	2	2	2	2	1	2	2
2	2	2	2	2	2	2	2	1
2	2	2	2	1	2	2	2	2
2	2	2	1	2	2	2	2	2
2	2	2	2	2	1	2	2	2
1	2	2	2	2	2	2	2	2
2	2	1	2	2	2	2	2	2
2	2	2	1	2	2	2	2	2
2	2	2	2	1	2	2	2	2
2	2	2	2	2	2	2	2	1
2	1	2	2	1	2	1	2	2

N_TEM_CC	EST_SEMP	SEM_OPÇ_	N_TEM_SU	N_MUDAN	N_TEM_DI	OUTROS_	number_of	barreira_ca
2	2	2	2	2	1	2	0	1
2	2	2	2	2	1	2	0	1
2	2	2	2	2	1	2	0	1
2	2	2	2	2	1	2	0	1
2	2	2	2	2	1	2	0	1
2	2	2	2	2	1	2	0	1
2	2	2	2	2	1	2	0	1
2	2	2	2	2	1	2	0	1
2	2	2	2	2	2	1	1	1
2	2	2	2	2	2	2	1	1
2	2	2	2	2	2	2	1	1
1	2	2	2	2	2	2	1	1
2	2	2	2	2	2	2	1	1
2	2	2	2	1	2	2	1	1
2	2	2	2	2	2	2	1	1
2	2	2	2	2	2	2	1	1
2	2	2	2	2	2	2	1	1
2	2	2	2	2	2	2	1	1
2	2	2	2	2	2	2	1	1
1	2	2	2	2	2	2	1	1
2	2	1	2	2	2	2	1	1
1	2	2	2	2	2	2	1	1
2	2	2	2	2	2	2	1	1
2	2	2	2	2	2	2	1	1
2	2	2	2	2	2	2	1	1
2	2	2	2	2	2	2	1	1
2	2	2	2	2	2	1	1	1
2	2	2	2	2	2	2	1	1
2	2	2	2	2	2	2	1	1
2	2	2	2	2	2	2	1	1
2	2	2	2	2	2	2	1	1
2	2	2	2	2	2	2	1	1
2	2	2	2	1	2	2	1	1
2	2	1	2	2	2	2	1	1
2	2	2	2	2	2	2	1	1
2	2	2	2	2	2	2	1	1
2	2	2	2	2	2	2	1	1
2	2	2	2	2	2	2	1	1
1	2	2	2	2	2	2	1	1
2	2	2	2	2	2	1	1	1
2	2	2	2	2	2	2	2	2
2	2	2	2	2	2	2	2	2
1	2	2	2	2	2	2	2	2
2	2	2	2	2	2	1	2	2
2	2	2	2	2	2	2	2	2
2	2	2	2	1	2	2	2	2

2	2	1	2	2	2	2	2	2
2	2	2	2	2	2	2	2	2
2	2	2	2	2	2	2	2	2
1	2	2	2	2	2	2	2	2
2	2	2	2	2	2	2	2	2
2	2	2	2	2	2	2	2	2
2	2	2	2	2	2	2	2	2
2	2	2	2	2	2	2	2	2
2	2	1	2	2	2	2	2	2
1	2	2	2	2	2	2	3	2
2	2	2	2	2	2	1	3	2
2	2	2	1	1	2	2	3	2
1	2	2	2	2	2	2	3	2
2	2	2	2	2	2	1	3	2
2	2	2	2	2	2	2	3	2
1	2	2	2	2	2	2	3	2
2	2	2	2	2	2	2	3	2
2	2	2	2	2	2	1	3	2
2	2	2	2	2	2	1	3	2
2	2	1	2	2	2	2	3	2
2	2	2	2	2	2	2	3	2
2	1	2	2	2	2	2	3	2
2	2	1	2	2	2	1	4	2
2	2	2	2	2	2	1	4	2
2	2	1	2	2	2	1	4	2
2	2	2	1	2	2	1	4	2
1	2	2	2	2	2	2	7	2

Q8	Q9a	Q9b	Q9c	Q9d	Q9e	Q9f	Q10	Q11
2	2	1	1	1	3	3	3	2
2	2	4	1	2	4	4	3	2
2	2	1	4	1	3	3	3	2
2	2	1	4	1	3	3	3	2
2	9	9	9	9	9	9	3	1
2	2	3	1	1	3	1	3	2
3	2	1	1	4	3	1	1	2
2	2	1	1	4	3	3	3	2
2	2	1	1	1	3	3	3	2
2	2	1	1	1	3	3	3	2
2	4	4	1	4	3	3	3	2
3	4	3	3	1	3	3	3	2
2	2	4	1	1	3	3	3	1
2	2	1	1	1	3	3	3	2
2	4	4	1	4	3	3	3	2
2	2	1	1	1	3	3	3	2
2	2	1	1	4	3	1	3	2
2	2	3	4	1	4	3	3	2
2	2	1	1	1	4	3	3	1
3	2	1	1	1	3	3	3	2
2	2	1	1	1	3	3	3	2
2	2	2	1	1	3	3	3	2
2	2	1	1	1	1	3	3	2
1	2	1	4	2	4	4	3	2
2	4	3	4	3	4	1	3	2
2	2	1	1	1	3	3	3	2
2	4	4	1	4	3	3	3	2
3	1	1	1	1	3	3	3	2
2	2	1	1	1	3	3	1	2
2	4	1	1	4	4	4	1	2
2	2	1	1	1	3	3	3	1
2	2	1	1	2	3	3	3	2
2	2	1	1	1	3	3	3	1
2	2	1	1	1	3	3	3	2
2	2	4	1	4	3	3	3	1
2	2	1	1	1	3	3	3	2
2	3	1	1	2	3	3	3	2
2	2	1	1	1	3	3	3	2
2	2	4	1	2	4	3	3	2
2	2	1	1	1	3	3	3	2
2	2	1	1	1	3	2	3	1
2	2	1	1	1	3	3	3	2
2	2	4	4	4	3	3	3	2
2	4	4	4	4	4	1	3	2
2	2	1	1	2	3	1	3	2
2	4	4	4	4	4	4	3	2

2	2	1	1	1	3	3	2	1
2	1	1	1	4	3	3	3	2
2	4	4	4	4	3	1	3	2
2	3	1	1	4	3	3	3	2
2	3	1	1	1	3	3	3	2
2	2	1	1	1	4	3	3	1
2	2	1	1	1	3	3	3	2
2	1	3	3	2	3	1	3	2
2	2	1	1	1	4	4	4	1
2	4	4	1	1	3	3	4	1
2	2	1	1	1	3	3	3	1
2	2	4	1	2	3	1	3	2
2	4	4	4	4	3	4	3	1
2	4	4	1	4	3	3	3	2
2	2	1	1	1	3	3	3	1
2	4	1	1	1	3	1	3	2
2	2	1	1	1	3	3	3	1
2	2	1	1	4	3	3	3	2
2	4	4	4	4	3	1	3	2
3	2	1	1	4	3	3	1	2
2	2	1	1	1	3	3	3	1
2	1	1	1	1	3	1	3	2
3	2	1	1	1	3	3	3	2
2	4	4	1	1	3	4	3	1
2	2	4	1	1	4	3	3	2
2	2	1	1	4	3	3	3	2
2	2	1	3	1	3	3	3	2

Q11.Cere_I	Q11.Verd_	Q11.Frutas	Q11.Leite_	Q11.Carne_	Q11.Feijõe	Q11.Gord_	Q11.Doce_	Q12
9	9	9	9	9	9	9	9	2
9	9	9	9	9	9	9	9	1
9	9	9	9	9	9	9	9	1
9	9	9	9	9	9	9	9	1
7	5	2	8	6	1	3	4	0
9	9	9	9	9	9	9	9	0
9	9	9	9	9	9	9	9	1
9	9	9	9	9	9	9	9	2
9	9	9	9	9	9	9	9	1
9	9	9	9	9	9	9	9	1
9	9	9	9	9	9	9	9	0
9	9	9	9	9	9	9	9	1
1	2	3	5	4	6	8	7	1
9	9	9	9	9	9	9	9	1
9	9	9	9	9	9	9	9	1
9	9	9	9	9	9	9	9	1
9	9	9	9	9	9	9	9	0
9	9	9	9	9	9	9	9	1
8	2	6	3	4	1	7	5	1
9	9	9	9	9	9	9	9	0
9	9	9	9	9	9	9	9	1
9	9	9	9	9	9	9	9	0
9	9	9	9	9	9	9	9	0
9	9	9	9	9	9	9	9	2
9	9	9	9	9	9	9	9	2
9	9	9	9	9	9	9	9	1
9	9	9	9	9	9	9	9	0
9	9	9	9	9	9	9	9	1
9	9	9	9	9	9	9	9	0
9	9	9	9	9	9	9	9	1
8	6	2	3	4	1	7	5	0
9	9	9	9	9	9	9	9	1
1	4	7	3	5	6	8	2	0
7	4	3	2	6	1	8	5	0
7	4	1	3	5	6	8	2	2
9	9	9	9	9	9	9	9	0
9	9	9	9	9	9	9	9	1
9	9	9	9	9	9	9	9	1
9	9	9	9	9	9	9	9	1
9	9	9	9	9	9	9	9	0
8	4	6	3	2	1	5	7	1
9	9	9	9	9	9	9	9	1
9	9	9	9	9	9	9	9	1
9	9	9	9	9	9	9	9	1
9	9	9	9	9	9	9	9	1
9	9	9	9	9	9	9	9	0

7	4	5	3	6	1	8	2	1
9	9	9	9	9	9	9	9	1
9	9	9	9	9	9	9	9	1
9	9	9	9	9	9	9	9	1
9	9	9	9	9	9	9	9	1
8	2	6	3	4	1	7	5	1
9	9	9	9	9	9	9	9	0
9	9	9	9	9	9	9	9	0
5	7	3	1	6	4	2	8	0
8	2	3	5	4	7	1	6	0
5	1	7	3	4	6	8	2	2
9	9	9	9	9	9	9	9	1
1	4	6	2	7	8	5	3	1
9	9	9	9	9	9	9	9	1
8	6	2	3	7	1	4	5	0
9	9	9	9	9	9	9	9	1
7	1	5	3	4	6	8	2	2
9	9	9	9	9	9	9	9	0
9	9	9	9	9	9	9	9	1
9	9	9	9	9	9	9	9	1
1	4	6	3	7	2	8	5	1
9	9	9	9	9	9	9	9	2
9	9	9	9	9	9	9	9	2
1	2	6	3	4	5	8	7	1
9	9	9	9	9	9	9	9	1
9	9	9	9	9	9	9	9	1
9	9	9	9	9	9	9	9	1

Q13	Q14	Q15	Q16	Q17	Q18	Q19	Q20	Q21
0	1	0	2	0	0	1	1	1
0	1	2	0	0	0	0	0	1
0	1	2	2	2	0	1	0	1
0	1	0	0	0	0	0	1	1
0	1	0	0	1	0	0	0	1
0	1	0	2	0	0	0	0	1
1	1	0	1	2	1	0	1	1
0	2	0	2	2	0	2	0	1
0	1	0	0	0	0	0	0	1
0	1	0	0	0	0	1	0	1
1	2	1	1	0	0	0	1	1
0	0	0	0	0	1	1	1	1
0	1	0	1	1	0	0	0	1
0	1	0	0	1	0	0	0	1
0	1	0	0	1	0	1	0	1
0	1	1	0	2	0	0	0	1
0	0	0	0	0	1	1	0	1
1	1	0	0	0	0	0	0	1
0	1	2	2	1	0	0	0	1
0	1	1	0	0	0	1	0	1
0	1	0	0	0	0	0	0	1
1	1	1	0	0	0	1	0	1
1	1	0	2	2	2	0	0	2
1	1	1	2	0	0	1	2	2
1	2	2	1	1	0	2	2	1
0	1	1	0	0	0	1	0	1
0	1	0	0	2	0	0	0	1
0	1	1	0	1	1	0	0	1
0	1	0	0	0	0	1	0	1
0	1	0	0	0	0	1	0	1
0	1	0	1	1	0	0	0	1
0	1	2	2	1	0	0	0	1
0	1	0	0	0	0	1	0	1
0	1	0	0	0	0	1	0	1
2	2	9	9	9	9	9	9	9
0	1	0	1	0	0	1	0	1
0	1	2	0	0	0	0	0	1
0	0	0	0	1	1	0	0	0
1	1	2	1	2	2	0	1	1
0	1	1	0	1	0	1	0	1
0	1	0	0	0	0	0	0	1
0	1	0	0	0	0	0	0	1
1	2	2	2	0	0	1	1	1
0	2	0	0	2	0	2	1	2
0	1	1	2	0	0	1	0	1
1	1	1	0	0	1	0	1	1

0	1	0	0	1	0	0	0	1
0	1	0	1	0	0	1	0	1
0	1	0	0	0	1	0	1	0
0	0	1	0	0	0	0	0	1
1	1	1	0	1	0	1	0	1
0	1	2	2	1	0	0	0	1
0	1	0	1	0	0	1	0	1
0	1	0	1	0	0	0	1	0
0	1	1	2	2	0	2	0	1
0	1	0	2	0	0	2	0	1
0	2	0	0	0	0	0	2	1
1	1	1	1	1	1	1	1	1
1	0	0	2	1	1	0	2	1
0	2	0	2	0	1	1	0	1
0	1	0	0	0	0	0	0	1
0	1	0	0	0	0	0	0	1
0	1	1	0	0	0	0	1	1
1	1	1	1	2	1	0	0	1
0	2	0	0	1	0	1	0	1
0	1	0	1	1	0	1	2	1
0	1	0	1	2	2	1	0	1
1	2	1	2	0	1	1	0	2
0	1	0	0	2	2	0	0	1
1	2	0	2	0	0	0	2	1
0	1	0	0	0	0	0	0	1
0	1	0	0	1	0	0	0	1
0	1	1	0	0	0	1	1	1

Nota_prop	Nota_prop	Nota_final_	Nota_prop	class_nk	Nota_prop	Nota_prop	Antilog_PT	Class_antil
100	60	15	57.69231	2	12.5	5750	0.680822	2
50	90	14	53.84615	2	12.5	5083.333	0.767134	2
87.5	60	14	53.84615	2	12.5	5333.333	1.05508	2
87.5	90	17	65.38462	1	12.5	6333.333	0.873587	2
25	80	12	46.15385	2	25	4333.333	1.958899	1
75	80	15	57.69231	2	12.5	5583.333	1.130089	2
50	50	10	38.46154	2	12.5	3750	1.349218	1
87.5	50	13	50	2	12.5	5000	0.607083	2
100	100	19	73.07692	1	12.5	7083.333	1.239249	1
100	90	18	69.23077	1	12.5	6750	0.977053	2
62.5	40	10	38.46154	2	12.5	3833.333	1.862884	1
50	60	11	42.30769	2	12.5	4083.333	1.814454	1
87.5	80	17	65.38462	1	25	6416.667	2.340967	1
100	90	18	69.23077	1	12.5	6750	3.314369	1
62.5	80	14	53.84615	2	12.5	5166.667	1.2681	1
100	80	17	65.38462	1	12.5	6416.667	1.216269	1
75	60	13	50	2	12.5	4916.667	0.951358	2
62.5	90	15	57.69231	2	12.5	5500	1.191526	2
87.5	70	17	65.38462	1	37.5	6500	1.821917	1
87.5	70	15	57.69231	2	12.5	5666.667	1.126075	2
100	100	19	73.07692	1	12.5	7083.333	1.724612	1
87.5	60	14	53.84615	2	12.5	5333.333	1.614651	1
87.5	40	12	46.15385	2	12.5	4666.667	1.307443	1
37.5	30	7	26.92308	2	12.5	2666.667	1.433625	1
25	20	5	19.23077	2	12.5	1916.667	1.310031	1
100	80	17	65.38462	1	12.5	6416.667	1.772169	1
62.5	80	14	53.84615	2	12.5	5166.667	1.146849	2
75	70	14	53.84615	2	12.5	5250	2.050075	1
87.5	80	16	61.53846	1	12.5	6000	1.613499	1
37.5	90	13	50	2	12.5	4666.667	1.5364	1
100	70	18	69.23077	1	37.5	6916.667	1.802533	1
87.5	70	15	57.69231	2	12.5	5666.667	1.839457	1
100	80	20	76.92308	1	37.5	7250	2.073591	1
100	80	18	69.23077	1	25	6833.333	1.796651	1
75	0	9	34.61539	2	25	3333.333	2.628498	1
100	70	16	61.53846	1	12.5	6083.333	1.352101	1
75	90	16	61.53846	1	12.5	5916.667	1.575987	1
100	60	15	57.69231	2	12.5	5750	0.810359	2
62.5	40	10	38.46154	2	12.5	3833.333	0.288149	2
100	60	15	57.69231	2	12.5	5750	1.640442	1
87.5	100	19	73.07692	1	25	7083.333	0.713755	2
100	100	19	73.07692	1	12.5	7083.333	1.238203	1
62.5	40	10	38.46154	2	12.5	3833.333	1.021799	2
25	50	8	30.76923	2	12.5	2916.667	1.273318	1
75	70	14	53.84615	2	12.5	5250	1.096334	2
25	50	8	30.76923	2	12.5	2916.667	1.737497	1

87.5	90	20	76.92308	1	37.5	7166.667	1.105014	2
75	80	15	57.69231	2	12.5	5583.333	1.288015	1
37.5	70	11	42.30769	2	12.5	4000	1.16594	2
75	80	15	57.69231	2	12.5	5583.333	1.888499	1
87.5	60	14	53.84615	2	12.5	5333.333	1.482896	1
87.5	70	17	65.38462	1	37.5	6500	1.258156	1
100	70	16	61.53846	1	12.5	6083.333	1.123402	2
37.5	60	10	38.46154	2	12.5	3666.667	0.561267	2
62.5	50	10	38.46154	2	0	3750	0.746538	2
62.5	70	13	50	2	12.5	4833.333	1.078198	2
100	70	20	76.92308	1	50	7333.333	3.313314	1
62.5	30	9	34.61539	2	12.5	3500	1.594125	1
37.5	40	7	26.92308	2	0	2583.333	1.185682	2
62.5	60	12	46.15385	2	12.5	4500	1.299265	1
100	90	19	73.07692	1	25	7166.667	1.732629	1
75	100	17	65.38462	1	12.5	6250	1.063566	2
100	70	19	73.07692	1	37.5	6916.667	1.019537	2
87.5	40	12	46.15385	2	12.5	4666.667	1.063974	2
37.5	70	11	42.30769	2	12.5	4000	1.88365	1
62.5	60	12	46.15385	2	12.5	4500	0.919566	2
100	60	16	61.53846	1	25	6166.667	1.084649	2
75	20	9	34.61539	2	12.5	3583.333	0.879201	2
87.5	70	15	57.69231	2	12.5	5666.667	1.551304	1
62.5	60	14	53.84615	2	37.5	5333.333	1.763952	1
75	100	17	65.38462	1	12.5	6250	1.825804	1
87.5	90	17	65.38462	1	12.5	6333.333	1.105436	2
87.5	70	15	57.69231	2	12.5	5666.667	1.920646	1

Antilog_CA	Class_antil	Antilog_LIP	Class_antil	Antilog_SA	Class_antil	Antilog_M(	Class_antil	Antilog_PO
4.514158	2	25.70835	1	1.117899	1	1.121974	2	0.551952
8.032848	1	14.30076	2	4.14223	1	3.661256	2	1.185886
2.596151	2	54.00378	1	4.997338	1	4.409846	2	2.704175
1.752284	2	24.06719	2	2.340159	1	1.722735	2	0.261294
3.021138	2	18.79374	2	8.588759	1	7.834477	2	2.286323
2.930565	2	38.29246	1	11.1416	2	10.77853	1	3.044751
2.747053	2	26.99646	1	6.727388	1	7.105076	2	2.249139
1.365683	2	7.482988	2	0.916868	1	0.913367	2	0.039149
3.637408	2	20.40764	2	5.662487	1	4.831768	2	2.202117
4.172545	2	19.67728	2	2.063222	1	2.082057	2	0.643967
4.636378	2	17.61534	2	3.858024	1	3.664421	2	1.95588
4.584999	2	20.56465	2	9.997961	2	9.966827	2	5.234706
3.31395	2	24.79353	2	5.742449	1	5.282223	2	3.101522
6.607119	1	40.24437	1	28.60023	2	22.78013	1	5.782948
4.101272	2	15.65755	2	10.16669	2	10.1118	1	3.945578
4.315168	2	17.81023	2	16.37271	2	6.474336	2	2.454203
4.791227	2	11.82425	2	6.497734	1	6.13237	2	3.064701
3.5789	2	28.77991	1	10.23877	2	10.24666	1	5.171415
3.71993	2	14.18796	2	3.095203	1	3.280729	2	1.413951
4.31061	2	25.46146	1	6.825901	1	7.64282	2	5.499118
5.162907	1	6.680323	2	4.634654	1	3.896872	2	2.018901
4.54044	2	17.97416	2	6.262594	1	7.589857	2	5.54237
4.895054	2	11.32321	2	8.987349	1	6.259258	2	3.630823
3.234131	2	32.81716	1	11.19059	2	13.31832	1	6.619334
4.147639	2	22.44183	2	8.507326	1	8.825107	2	4.394609
3.827306	2	22.79017	2	8.680696	1	9.614268	2	7.033662
4.547871	2	28.21413	1	9.703213	1	6.487804	2	2.334256
3.547981	2	18.67406	2	6.946063	1	6.944308	2	4.945053
4.284566	2	26.87149	1	11.70693	2	11.69748	1	3.861175
3.596852	2	27.75803	1	11.55168	2	12.49877	1	5.682355
3.460105	2	28.98514	1	2.303104	1	2.545583	2	1.365686
3.727094	2	31.21227	1	3.130905	1	3.620499	2	1.61452
3.46969	2	37.8417	1	19.04051	2	16.6423	1	2.848373
2.330762	2	22.98202	2	8.018936	1	8.258817	2	3.030108
4.609392	2	49.15951	1	19.48364	2	21.414	1	11.98786
4.145324	2	31.25316	1	12.61407	2	12.17732	1	4.359881
4.706951	2	25.60216	1	9.647453	1	6.765179	2	3.289838
2.530622	2	12.22841	2	8.964083	1	9.39554	2	5.443446
3.190193	2	8.432214	2	7.398636	1	7.063024	2	2.971028
4.542214	2	13.35399	2	2.534997	1	2.87378	2	1.221899
2.451628	2	53.08045	1	10.83266	2	8.019921	2	3.048966
4.650558	2	20.10696	2	5.249947	1	5.103433	2	2.964403
4.426296	2	17.38235	2	6.035485	1	6.078627	2	1.810144
4.314427	2	23.44008	2	11.30177	2	9.548094	2	3.893431
3.440234	2	21.76721	2	11.3802	2	7.341007	2	1.534089
3.610446	2	19.35358	2	8.941783	1	7.243579	2	4.196508

3.416676	2	36.12153	1	2.644051	1	1.660994	2	0.23617
2.559678	2	24.84499	2	11.09263	2	12.52931	1	6.109006
4.33898	2	29.60598	1	12.03276	2	12.03383	1	5.019511
2.845078	2	25.68333	1	14.10217	2	12.19727	1	2.746805
3.865368	2	28.85918	1	9.142125	1	11.17943	1	6.135814
7.100031	1	23.75419	2	4.748424	1	5.656505	2	4.586109
4.631125	2	37.37954	1	12.12673	2	9.4113	2	3.834211
4.849966	2	17.51705	2	7.02392	1	6.420193	2	4.796019
3.221108	2	36.69012	1	12.33548	2	9.234853	2	3.008475
4.492644	2	19.17685	2	2.673233	1	2.745039	2	1.017862
5.471176	1	26.95362	1	12.37914	2	11.62113	1	4.251912
4.771622	2	19.9666	2	7.634707	1	8.575948	2	4.677065
3.719128	2	32.03849	1	12.43	2	11.40728	1	3.154744
3.477981	2	29.69919	1	13.30157	2	12.33083	1	5.577004
4.905659	2	24.0404	2	6.279244	1	7.811827	2	1.99973
3.545296	2	21.93292	2	7.120852	1	8.142666	2	5.123773
3.979264	2	23.46066	2	5.497956	1	3.776705	2	0.693208
3.017703	2	29.07763	1	3.578538	1	3.32656	2	1.088067
3.219041	2	35.0861	1	12.2626	2	13.51073	1	2.481526
5.018942	1	19.16547	2	7.951966	1	7.950402	2	3.965734
3.947963	2	27.78569	1	4.834056	1	4.987365	2	4.351358
3.927537	2	20.45414	2	8.055733	1	7.765709	2	2.61694
4.075711	2	18.75316	2	7.817097	1	7.811196	2	4.837497
3.760215	2	36.97089	1	11.6268	2	13.76565	1	4.329544
3.370865	2	18.06194	2	10.23445	2	8.353942	2	4.510405
2.761581	2	49.8165	1	10.4207	2	11.6341	1	8.630437
2.488095	2	24.70567	2	10.70782	2	11.67099	1	2.886814

Class_antil	Antilog_FIE	Class_antil	Antilog_CO	Class_antil	Antilog_vit	Class_antil	Antilog_vit	Class_antil
2	11.99883	2	1.572436	1	0.980154	2	1.431117	1
2	5.790523	2	167.7952	1	0.574217	2	0.963044	2
2	11.32919	2	286.9047	1	0.59927	2	1.69117	1
2	5.706863	2	12.68623	1	0.738217	2	2.068948	1
2	5.706863	2	586.3756	2	0.7929	2	0.863561	2
2	7.887659	2	377.9077	2	0.220291	2	0.48754	2
2	6.30022	2	167.5392	1	1.112027	1	0.654059	2
2	4.427289	2	50.94897	1	0.19803	2	0.314854	2
2	7.913392	2	110.5144	1	1.003776	1	1.22805	1
2	8.721999	2	122.3169	1	1.349581	1	1.039949	2
2	7.377135	2	230.8502	1	0.651206	2	0.997624	2
2	14.19352	2	543.3449	2	1.110389	1	2.188489	1
2	6.176803	2	321.9111	2	0.7395	2	0.810851	2
2	7.376242	2	955.6326	2	2.689318	1	3.534853	1
2	11.26127	2	297.4323	1	1.11391	1	1.702728	1
2	11.34648	2	546.8501	2	1.117185	1	4.189278	1
2	11.64145	2	136.0478	1	0.766652	2	0.520775	2
2	26.432	1	331.8989	2	1.094439	1	1.573987	1
2	3.622777	2	305.1053	2	0.891481	2	0.455035	2
2	6.30022	2	101.7247	1	0.671781	2	0.628337	2
2	4.509853	2	293.6188	1	0.906763	2	2.314012	1
2	8.660008	2	416.3372	2	1.234817	1	2.741693	1
2	9.636214	2	326.655	2	1.739271	1	2.367696	1
1	4.043381	2	343.7054	2	0.857488	2	1.054274	2
2	12.50646	2	432.4432	2	1.03746	1	2.587218	1
1	14.0681	2	381.914	2	0.638522	2	0.631244	2
2	8.075633	2	207.0327	1	0.587825	2	0.448549	2
2	13.89157	2	486.0197	2	1.456275	1	2.600726	1
2	23.69928	1	439.7573	2	1.555706	1	1.768528	1
2	21.36879	2	475.3562	2	1.651169	1	2.540799	1
2	6.740238	2	221.5879	1	0.801262	2	1.636883	1
2	7.376242	2	288.2429	1	0.896439	2	1.508204	1
2	3.622777	2	590.9334	2	1.049748	1	0.886432	2
2	11.61775	2	696.3305	2	0.678242	2	0.922429	2
1	3.600468	2	1626.083	2	1.474097	1	2.179571	1
2	8.475335	2	218.0685	1	0.967595	2	0.58206	2
2	14.86533	2	385.8761	2	1.004912	1	2.342406	1
2	5.540188	2	859.7545	2	1.39034	1	5.825832	1
2	6.813991	2	379.3927	2	1.269103	1	1.782521	1
2	7.887659	2	193.1282	1	1.242198	1	0.835783	2
2	5.43275	2	246.2947	1	1.844884	1	1.466071	1
2	9.582567	2	175.0322	1	0.920261	2	1.306644	1
2	9.595442	2	91.49778	1	1.350207	1	1.170743	1
2	11.43484	2	198.3917	1	1.418151	1	0.832045	2
2	2.979339	2	263.7062	1	0.728002	2	1.19877	1
2	9.384359	2	374.1748	2	1.03015	1	1.165194	1

2	7.967519	2	74.37053	1	0.556696	2	1.897584	1
1	8.144694	2	437.0579	2	1.279388	1	1.42245	1
2	18.43762	2	323.0874	2	1.141843	1	1.558428	1
2	17.96896	2	297.2184	1	1.437323	1	1.543094	1
1	12.70393	2	325.8167	2	1.051138	1	1.145709	1
2	11.61775	2	173.6208	1	0.825207	2	0.899554	2
2	12.25957	2	124.1324	1	1.395695	1	0.863286	2
2	19.38983	2	223.1548	1	1.233532	1	0.875306	2
2	9.75701	2	146.7804	1	1.290577	1	0.806555	2
2	4.427289	2	94.97011	1	0.929463	2	0.485775	2
2	7.377135	2	768.7302	2	1.258634	1	1.285826	1
2	13.04406	2	329.4324	2	0.886498	2	0.929696	2
2	21.1128	2	510.3892	2	1.178457	1	1.865668	1
2	15.51112	2	362.6453	2	1.915825	1	1.727743	1
2	6.740238	2	312.1268	2	1.036371	1	0.687355	2
2	23.19453	1	307.3074	2	1.195773	1	1.842605	1
2	5.566751	2	167.7668	1	0.765648	2	0.818913	2
2	3.600468	2	233.0456	1	0.82023	2	1.189701	1
2	12.14366	2	309.6474	2	1.005272	1	1.488726	1
2	17.50536	2	392.0724	2	1.299717	1	2.181906	1
2	11.70594	2	183.5686	1	0.861985	2	1.159464	1
2	15.42133	2	1044.301	2	1.242806	1	1.817495	1
2	20.97937	2	280.4234	1	1.043494	1	1.211374	1
2	21.88774	1	609.3935	2	1.038306	1	1.38816	1
2	18.07953	2	284.649	1	1.274956	1	1.635141	1
1	39.50641	1	548.0178	2	0.770722	2	1.232123	1
2	14.9914	2	512.4096	2	1.903565	1	1.618294	1

Antilog_vit	Class_antil	Antilog_vit	Class_antil	Antilog_vit	Class_antil	Antilog_vit	Class_antil	Antilog_vit
8.719602	2	0.455122	2	4.497616	1	38.76894	2	8.958038
45.91944	1	0.18602	2	1.52956	2	41.54406	2	1.283723
44.2759	1	1.207657	1	9.951116	1	36.04144	2	4.437706
3.116502	2	0.851387	2	3.966418	1	0.874594	2	5.387265
3.116502	2	0.188709	2	1.499186	2	7.958809	2	1.506608
55.57457	1	0.420584	2	1.744929	2	8.252705	2	1.553411
21.35859	1	0.451582	2	0.637578	2	8.1033	2	0.793139
20.06367	1	0.167783	2	1.769443	2	0.523551	2	0.966957
14.46642	1	0.245997	2	3.057446	1	19.24733	2	1.681492
28.86405	1	0.452691	2	2.291809	1	4.338284	2	1.636004
14.97968	1	0.556283	2	6.154829	1	7.063139	2	1.256333
45.3146	1	1.711524	1	3.220012	1	58.31318	2	4.348732
70.60901	1	0.558604	2	6.251288	1	63.04421	1	5.864018
60.30717	1	1.965674	1	5.849898	1	139.8795	1	3.783133
37.9913	1	1.054115	2	2.112793	1	552.3447	1	3.32587
82.82957	1	1.988037	1	96.17686	1	64.75834	1	4.827303
31.9363	1	0.702535	2	1.164388	2	19.85735	2	7.063499
60.95537	1	1.657444	1	4.823715	1	32.5805	2	3.648745
55.88877	1	0.196582	2	2.572371	1	23.16695	2	1.212051
21.35859	1	0.345528	2	0.83204	2	14.59267	2	1.07461
41.16733	1	0.796058	2	32.25872	1	13.77937	2	3.105808
65.63918	1	1.456391	1	48.54356	1	49.56045	2	14.50837
53.33022	1	1.057104	2	38.13013	1	40.62505	2	12.60622
40.1148	1	1.02496	2	4.022547	1	28.3641	2	14.23154
84.02295	1	1.57991	1	56.24837	1	33.33961	2	9.275079
57.39942	1	0.962924	2	1.481068	2	5.956755	2	22.03105
44.41004	1	0.867283	2	2.257242	1	32.62297	2	6.314706
45.16985	1	2.417365	1	10.93711	1	26.40751	2	5.508023
57.24997	1	1.190734	1	4.706403	1	79.16708	1	2.962425
69.90554	1	1.458692	1	21.29827	1	42.1795	2	3.919034
30.48035	1	0.761959	2	2.37848	1	1.471001	2	2.988491
60.30717	1	0.569912	2	7.939695	1	2.834198	2	3.291775
55.88877	1	0.335786	2	2.042578	1	21.25182	2	1.693062
26.67558	1	0.201094	2	1.763018	2	9.70236	2	1.868
25.54261	1	0.458332	2	5.795778	1	14.75277	2	3.759557
45.89703	1	0.70803	2	2.051821	1	38.46594	2	7.08627
86.78397	1	0.981249	2	42.52033	1	30.79518	2	7.941743
124.4852	1	2.912811	1	134.3061	1	50.28216	2	15.06107
127.9738	1	2.145923	1	29.62892	1	51.56781	2	7.372738
55.57457	1	0.204284	2	3.257831	1	2.415438	2	1.250276
57.09605	1	1.496269	1	3.529123	1	123.5771	1	2.860101
30.56173	1	0.445368	2	1.896644	2	52.04041	2	2.753331
23.423	1	0.553481	2	0.908724	2	4.786269	2	0.945301
51.07549	1	0.697111	2	1.637033	2	28.08751	2	9.732576
50.99119	1	0.832932	2	19.54972	1	18.66564	2	3.764367
102.4046	1	1.451593	1	2.684156	1	16.51117	2	10.27538

11.91013	2	0.539528	2	6.848986	1	3.957707	2	4.78002
62.84448	1	1.26099	1	4.761586	1	31.20287	2	18.02244
50.70482	1	1.440963	1	1.617877	2	25.44014	2	3.374292
62.64134	1	1.614693	1	4.88473	1	74.96361	1	2.269488
79.11571	1	1.577417	1	4.98379	1	141.6842	1	4.210049
26.67558	1	0.37607	2	2.73228	1	80.53781	1	2.320443
34.21101	1	0.917204	2	3.759888	1	48.58589	2	7.569907
55.90609	1	0.994315	2	2.794179	1	28.72091	2	12.63971
59.32668	1	1.397174	1	2.508588	1	53.19166	2	10.48718
20.06367	1	0.151803	2	0.760363	2	50.31323	2	1.160348
14.97968	1	0.650851	2	1.87144	2	57.75825	2	1.857187
46.41198	1	1.252322	1	3.347873	1	160.6525	1	3.802282
65.898	1	1.933355	1	6.571144	1	248.4736	1	5.978911
66.89591	1	1.230391	1	2.02051	1	59.53598	2	3.004864
30.48035	1	0.28639	2	1.561365	2	46.18945	2	1.342612
56.77722	1	1.896737	1	4.238171	1	4338.221	1	3.731835
27.67725	1	0.244529	2	4.90916	1	122.4358	1	1.676181
25.54261	1	0.665067	2	9.795127	1	2.30512	2	2.64348
76.57987	1	1.81268	1	9.239329	1	12.70977	2	7.509056
33.93426	1	1.636165	1	2.396207	1	78.42778	1	3.412129
50.36159	1	0.509389	2	2.504816	1	8.969463	2	2.338175
53.22614	1	0.969555	2	42.48298	1	51.57796	2	5.135461
59.49761	1	1.439439	1	6.161118	1	82.28014	1	4.153959
62.47081	1	1.522136	1	10.79225	1	136.2519	1	6.953892
53.16558	1	1.750714	1	6.831163	1	139.9098	1	6.608965
74.28713	1	2.902341	1	8.044813	1	153.466	1	5.853634
98.41136	1	1.583443	1	5.281133	1	101.466	1	3.046645

Class_antil	Antilog_FO	Class_antil	Antilog_CA	Class_antil	Antilog_FO	Class_antil	Antilog_FE	Class_antil
2	92.52639	2	264.0631	2	756.2694	2	12.26867	1
2	6.104626	2	284.3899	2	818.4717	2	8.320488	1
2	110.705	2	257.4096	2	880.5041	2	9.572043	1
2	127.1951	2	379.7262	2	854.5041	2	7.437614	2
2	30.36837	2	372.7748	2	1612.319	1	18.02915	1
2	20.26608	2	91.94454	2	684.705	2	6.277997	2
2	2.989787	2	159.2236	2	808.967	2	8.334588	1
2	11.36875	2	93.5436	2	384.4838	2	7.136802	2
2	31.41427	2	412.0609	2	1066.76	1	12.27593	1
2	24.95281	2	270.5633	2	896.3759	2	11.79758	1
2	25.18933	2	181.7974	2	1100.902	1	11.82371	1
2	160.0858	2	494.7736	2	1163.341	1	12.35829	1
2	26.14425	2	325.4683	2	1114.431	1	11.15649	1
2	224.9355	2	1197.686	1	2409.529	1	19.23011	1
2	161.5018	2	532.7129	2	1107.139	1	12.58283	1
2	233.0525	2	260.8608	2	1046.143	2	15.43251	1
2	30.87243	2	235.9813	2	845.0786	2	12.52398	1
2	183.8367	2	182.2805	2	863.6323	2	10.87978	1
2	5.381544	2	152.4448	2	1220.211	1	6.929329	2
2	10.07336	2	138.2933	2	764.1387	2	8.180789	1
2	90.33839	2	243.5362	2	1187.459	1	14.63148	1
1	151.4855	2	230.4206	2	1113.291	1	15.61232	1
1	128.8789	2	339.6195	2	1129.248	1	22.22296	1
1	52.00067	2	282.0228	2	939.4838	2	11.02996	1
2	143.248	2	218.1443	2	991.3247	2	13.65318	1
1	38.43393	2	143.0221	2	1022.403	2	12.34985	1
2	37.04542	2	224.8265	2	511.5442	2	6.930846	2
2	157.6279	2	386.1252	2	1635.579	1	8.602986	1
2	175.5246	2	345.9067	2	1105.717	1	10.44772	1
2	299.4166	2	498.3792	2	1527.994	1	13.36343	1
2	82.88437	2	154.9566	2	1077.892	1	13.32486	1
2	75.54148	2	197.1828	2	1080.169	1	14.27164	1
2	30.65853	2	501.4154	2	1760.172	1	14.152	1
2	46.59075	2	246.9456	2	1313.966	1	15.35275	1
2	123.5673	2	427.6338	2	2153.381	1	23.44651	1
2	38.82722	2	153.1798	2	831.1941	2	11.59751	1
2	119.835	2	511.4052	2	1182.873	1	12.00858	1
1	304.2139	2	259.174	2	1560.138	1	20.78884	1
2	106.1637	2	161.6994	2	1135.638	1	14.21862	1
2	10.85115	2	183.6727	2	1112.502	1	15.64047	1
2	47.68317	2	705.196	2	1165.457	1	19.78237	1
2	54.79459	2	339.8182	2	1031.751	2	13.57161	1
2	4.620549	2	203.8932	2	952.8452	2	9.103849	1
2	68.10686	2	292.069	2	901.7673	2	14.27743	1
2	55.40449	2	185.3054	2	752.1011	2	9.859146	1
2	58.05705	2	460.3023	2	1277.249	1	10.83538	1

2	122.566	2	307.6848	2	969.1836	2	9.776105	1
1	93.03928	2	361.8451	2	1129.656	1	16.73042	1
2	134.0982	2	253.2516	2	748.6837	2	9.725683	1
2	48.19597	2	728.9143	2	1320.589	1	11.88008	1
2	318.2759	2	192.7879	2	844.1641	2	12.0399	1
2	45.0276	2	208.4611	2	1120.208	1	10.62176	1
2	61.50658	2	371.8149	2	499.624	2	8.398802	1
1	59.43544	2	472.1484	2	952.1164	2	12.24158	1
2	117.5428	2	202.819	2	612.612	2	13.81795	1
2	13.44806	2	194.8029	2	886.7671	2	9.154537	1
2	39.51046	2	1056.754	2	2506.159	1	16.93145	1
2	118.8719	2	193.9322	2	884.9716	2	9.668872	1
2	291.3844	2	252.5906	2	868.2709	2	11.85322	1
2	147.6041	2	448.2513	2	1152.8	1	9.148924	1
2	4.04314	2	229.4798	2	1076.494	1	15.14905	1
2	177.6479	2	211.6745	2	855.3411	2	10.99814	1
2	12.91453	2	260.0509	2	936.4892	2	10.91056	1
2	46.61454	2	175.6682	2	838.0392	2	11.14073	1
2	80.56376	2	165.6068	2	1039.031	2	15.04197	1
2	144.0755	2	223.9886	2	816.7679	2	6.846734	2
2	39.3332	2	299.8426	2	1026.085	2	9.397063	1
2	112.3166	2	182.2566	2	774.2912	2	13.31347	1
2	152.7775	2	308.6047	2	1187.505	1	9.318574	1
2	312.4735	2	202.5038	2	1020.043	2	13.77229	1
2	201.1423	2	868.6216	2	1666.318	1	14.57719	1
2	138.9476	2	184.9046	2	916.6024	2	7.428074	2
2	94.35495	2	151.8494	2	1336.374	1	11.93407	1

Antilog_ZIN	Class_antilo	Antilog_SÓ	Class_antilo	Antilog_MA	Class_antilo	Antilog_Ce	Class_cerea	Antilog_Fru
1.649929	2	366.6994	1	143.5117	2	4.435088	2	4.968214
3.344368	2	327.9055	1	45.70137	2	6.417718	1	2.325938
7.290791	2	1571.277	2	94.98582	2	3.033961	2	2.267895
4.557259	2	557.3674	1	88.28888	2	1.406393	2	0.98799
22.45399	1	498.9464	1	111.255	2	5.911958	2	2.588535
9.754243	1	851.2618	1	81.55793	2	5.117857	2	2.532738
7.251451	2	1163.156	1	61.6182	2	4.365486	2	1.448186
3.502424	2	86.14601	1	18.24295	2	3.867302	2	1.311213
6.778986	2	598.3107	1	92.00051	2	3.263564	2	1.461474
4.008617	2	767.4901	1	55.26347	2	8.684707	1	4.889465
8.203844	2	950.6967	1	103.4293	2	5.404295	2	1.315369
11.57629	1	1037.518	1	231.2269	2	4.270115	2	4.882182
6.316089	2	1413.65	1	129.3869	2	4.787305	2	2.605503
21.50482	1	3889.938	2	275.8157	2	8.163074	1	4.65385
11.18132	1	1394.344	1	199.3437	2	5.18794	2	5.027346
9.766202	1	1640.699	2	178.6705	2	2.477963	2	4.088522
7.324883	2	1331.274	1	183.4634	2	3.555594	2	4.599037
10.23364	1	2498.601	2	201.5223	2	8.981915	1	1.011819
4.667106	2	254.75	1	80.86056	2	6.189116	1	2.53795
3.181479	2	857.5724	1	72.93216	2	16.29604	1	2.72259
7.617571	2	582.8753	1	72.90685	2	4.121705	2	0.889437
8.577197	1	1524.278	2	125.1376	2	4.19091	2	3.564745
6.904004	2	1637.057	2	130.8869	2	1.887542	2	2.225145
9.431655	1	1540.988	2	119.3013	2	1.436133	2	2.696901
8.089083	2	2120.903	2	179.7291	2	6.697758	1	2.987961
9.616271	1	2430.729	2	163.7995	2	3.258685	2	0.977579
5.140642	2	1934.336	2	90.35553	2	2.048255	2	5.375122
8.822823	1	2062.625	2	342.504	1	6.000857	1	1.703426
12.40626	1	2625.071	2	214.5268	2	5.447019	2	3.022555
11.94643	1	2426.993	2	232.3782	2	6.178392	1	1.677884
9.444588	1	695.6231	1	72.85902	2	5.517248	2	1.035413
8.235906	2	437.8778	1	64.70465	2	4.887119	2	1.052907
15.19523	1	955.1561	1	77.64908	2	6.861027	1	2.007936
18.07646	1	259.9599	1	84.26781	2	4.284876	2	1.797986
24.78146	1	1271.254	1	185.8034	2	6.731087	1	1.42324
8.345208	2	1600.799	2	175.4886	2	3.165626	2	3.34799
7.76874	2	1780.438	2	192.0223	2	4.591697	2	2.96842
13.45702	1	2513.15	2	131.1209	2	3.904535	2	2.692805
6.56224	2	2131.896	2	145.6958	2	2.7239	2	2.657857
5.020548	2	126.6705	1	34.35603	2	6.896922	1	1.068022
8.401443	2	1466.342	1	70.50584	2	3.037407	2	1.999331
5.00323	2	605.2163	1	78.09317	2	20.49905	1	1.222751
3.244143	2	516.2118	1	119.4549	2	4.787981	2	1.875924
5.365769	2	1392.729	1	136.3672	2	5.372481	2	3.089318
3.898027	2	1046.471	1	71.86241	2	4.391737	2	3.172809
6.269637	2	1261.444	1	203.605	2	2.905377	2	0.979266

5.316493	2	682.3352	1	77.91471	2	5.354866	2	1.53738	
15.37057	1	1296.052	1	131.4451	2	5.681558	2	2.597852	
7.292396	2	2552.536	2	204.5161	2	4.972911	2	2.092839	
17.30758	1	1216.472	1	152.6001	2	4.224427	2	2.298266	
12.05736	1	803.525	1	231.9089	2	4.775064	2	3.356083	
6.720735	2	495.1702	1	134.5936	2	6.141028	1	3.949673	
6.69209	2	1885.008	2	158.5657	2	2.467211	2	5.368395	
5.956979	2	2115.559	2	236.991	2	5.705833	2	3.131991	
5.562822	2	1937.93	2	159.0926	2	3.690478	2	2.248293	
3.580256	2	649.2243	1	61.56585	2	7.92008	1	2.688459	
16.54789	1	1327.739	1	253.6131	2	7.930809	1	5.959632	
9.971709	1	1259.475	1	235.6299	2	6.799801	1	4.064302	
11.37508	1	1525.245	2	227.9624	2	5.646912	2	3.521194	
8.78398	1	2600.9	2	179.5195	2	4.373927	2	3.801871	
12.61863	1	660.1745	1	109.9734	2	6.07862	1	3.737841	
10.31065	1	2010.058	2	248.616	2	6.21769	1	5.345368	
4.048652	2	609.6701	1	44.55578	2	5.398354	2	2.979571	
7.46209	2	546.6118	1	68.64531	2	5.280867	2	1.12134	
16.46627	1	2070.785	2	152.2371	2	4.146877	2	2.738882	
5.281531	2	2889.169	2	158.0753	2	6.859665	1	3.130598	
4.179734	2	410.1561	1	118.3754	2	6.112191	1	2.184642	
6.786715	2	2549.652	2	167.3917	2	3.783807	2	4.934099	
8.736949	1	1670.638	2	269.5723	2	5.375238	2	4.280418	
16.63985	1	1289.607	1	244.3344	2	4.52548	2	4.481993	
11.91272	1	1715.348	2	371.0051	1	5.460334	2	3.762396	
6.444114	2	1040.089	1	313.338	2	2.371343	2	3.786517	
20.45752	1	833.0763	1	228.84	2	3.840801	2	3.709931	

Class_fruta	Antilog_Ho	Class_horta	Antilog_Ca	Class_carne	Antilog_Lei	Class_leites	Antilog_Leg	Class_legur
1	2.32323	2	2.673395	1	1.234442	2	2.894623	1
2	1.755156	2	2.17911	1	2.147813	1	1.694682	2
2	0.910519	2	4.418583	2	1.856191	2	1.906576	2
2	2.720796	2	4.242275	2	1.990759	2	1.704902	2
2	1.013962	2	4.796732	2	1.939019	2	4.001757	1
2	0.980661	2	4.330141	2	1.066716	2	1.159405	2
2	1.634628	2	4.241583	2	1.750213	2	2.66543	1
2	1.069253	2	2.065827	1	1.172708	2	1.882727	2
2	1.713749	2	1.335292	1	2.476114	1	1.86199	2
1	1.297702	2	2.36938	1	1.489056	2	1.839748	2
2	0.986604	2	2.942314	1	1.259336	2	3.993783	1
1	1.615828	2	3.351203	2	1.836672	2	1.717061	2
2	0.966818	2	3.394149	2	1.626227	2	2.529921	1
1	3.694813	1	6.490408	2	5.423844	1	2.283843	1
1	1.978865	2	3.166386	2	1.688666	2	2.441664	1
1	1.830995	2	3.359128	2	1.920647	2	2.412324	1
1	1.067899	2	5.69349	2	2.631445	1	6.963206	1
2	1.825223	2	5.487648	2	1.233809	2	2.134733	1
2	0.97835	2	3.751761	2	1.075071	2	2.407516	1
2	1.041165	2	8.869003	2	1.675741	2	0.965736	2
2	1.143911	2	3.893154	2	2.038522	1	3.338594	1
1	2.29219	2	5.797669	2	2.747363	1	0.968933	2
2	1.059455	2	4.430889	2	2.83363	1	0.951309	2
2	1.005576	2	7.437018	2	1.276313	2	1.74161	2
1	1.677724	2	3.297476	2	2.25532	1	2.290846	1
2	1.026364	2	5.71709	2	0.91754	2	3.177721	1
1	0.981849	2	2.342442	1	1.062453	2	2.17415	1
2	1.00443	2	3.605012	2	1.281135	2	3.466731	1
2	1.014229	2	4.544676	2	1.565131	2	2.815409	1
2	1.021996	2	5.801206	2	1.563362	2	3.788092	1
2	0.960852	2	4.366693	2	1.141189	2	2.812933	1
2	1.51723	2	4.232848	2	1.216109	2	2.903639	1
2	0.97835	2	6.973653	2	1.838372	2	3.006338	1
2	1.561303	2	5.038135	2	1.243835	2	3.498227	1
2	0.917214	2	7.716906	2	1.996209	1	4.234758	1
1	3.101484	1	4.179061	2	4.006164	1	3.177521	1
1	1.447472	2	5.621277	2	2.641744	1	4.730092	1
2	1.007331	2	4.603386	2	0.976132	2	7.959591	1
2	1.022545	2	5.003025	2	0.92892	2	4.423928	1
2	0.980661	2	2.540456	1	1.066716	2	6.830929	1
2	2.255552	2	3.296385	2	3.336046	1	1.062938	2
2	1.025615	2	2.335479	1	0.919758	2	2.397906	1
2	1.008616	2	2.298079	1	0.972028	2	1.472641	2
1	1.022573	2	6.104973	2	3.826806	1	6.199238	1
1	1.060701	2	4.666422	2	3.637325	1	2.005223	1
2	2.499376	2	3.821968	2	2.927704	1	4.544576	1

2	0.953579	2	3.734082	2	1.712512	2	1.633758	2
2	1.049694	2	7.190696	2	0.851809	2	2.6467	1
2	0.998443	2	3.13669	2	1.547954	2	3.775079	1
2	1.035899	2	5.095755	2	3.381636	1	2.318245	1
1	1.437076	2	3.19653	2	1.030053	2	2.47911	1
1	1.020459	2	3.647797	2	0.935214	2	3.7036	1
1	0.947087	2	2.638181	1	1.196966	2	3.280577	1
1	0.988287	2	5.049701	2	3.898998	1	3.212547	1
2	3.534023	1	5.038561	2	3.83522	1	3.40258	1
2	1.069253	2	1.948316	1	1.083036	2	2.471451	1
1	2.496107	2	6.252222	2	2.091225	1	1.98298	2
1	1.027067	2	3.559109	2	0.979547	2	2.1498	1
1	1.842556	2	3.878214	2	1.068796	2	2.319991	1
1	1.29739	2	3.139307	2	2.09706	1	2.369353	1
1	2.411738	2	4.127634	2	1.517782	2	3.136407	1
1	0.990266	2	3.660595	2	1.032877	2	2.833856	1
1	0.931154	2	2.234137	1	2.042392	1	2.706199	1
2	0.917214	2	3.3422	2	1.798584	2	1.766961	2
2	0.987908	2	4.097197	2	1.041052	2	4.830523	1
1	1.003447	2	2.09516	1	1.15676	2	1.794654	2
2	0.960892	2	3.389446	2	1.542103	2	3.355109	1
1	0.989751	2	3.283846	2	1.034658	2	3.268978	1
1	1.550355	2	4.597138	2	1.206114	2	2.619903	1
1	0.968829	2	4.491654	2	1.110408	2	1.849945	2
1	5.116621	1	3.976994	2	2.661314	1	2.779203	1
1	1.296717	2	4.236444	2	1.846808	2	2.891943	1
1	1.015502	2	6.175894	2	1.168988	2	2.447245	1

Antilog_Óle	Class_óleos	Antilog_Do	Class_doce	Antilog_vit	VAR00002	adeq_2gru	ref_final
1.510733	2	2.578793	2	498.5896	2	2	5
1.279887	2	2.271239	2	123.7693	2	2	4.666667
1.835476	2	2.028421	2	929.8628	1	2	3.333333
0.914091	1	2.065108	2	867.5318	1	2	3
0.914091	1	0.936634	1	217.4535	2	2	3
1.134872	2	1.096589	2	94.94684	2	2	3
1.801836	2	1.793672	2	202.8642	2	2	3
0.648038	1	1.292213	2	34.28636	2	2	2.666667
4.955972	2	2.722345	2	161.0313	2	2	6
0.88563	1	1.16489	2	223.0035	2	2	6
2.534667	2	2.666715	2	104.8065	2	2	5
1.671135	2	8.377502	2	275.3406	2	2	4.666667
2.887537	2	1.670188	2	152.2936	2	2	4.666667
1.467093	2	2.869262	2	1526.977	1	1	4.666667
2.625361	2	6.118637	2	152.015	2	2	4.333333
3.065615	2	2.291444	2	8969.404	1	2	4.333333
2.515509	2	1.701315	2	121.6338	2	1	4.333333
3.426625	2	2.738928	2	851.959	1	2	4
1.152351	2	1.518157	2	293.1037	2	2	4
1.737385	2	0.826577	1	251.4031	2	2	4
0.782606	1	0.530035	1	2679.174	1	2	4
4.04139	2	1.338207	2	4805.945	1	1	4
2.276827	2	1.606415	2	3641.174	1	2	4
4.186442	2	1.675433	2	156.8631	2	2	4
3.076638	2	1.497028	2	5332.883	1	1	4
3.818758	2	4.749151	2	161.7252	2	2	3.666667
3.817154	2	1.090338	2	351.7373	2	2	3.333333
4.421554	2	1.204597	2	187.1305	2	2	3
1.569548	2	1.552875	2	201.6674	2	2	3
1.806535	2	0.902374	1	3002.077	1	2	3
3.016692	2	1.207498	2	401.3931	2	2	3
1.467093	2	1.32224	2	290.3454	2	2	3
1.152351	2	1.108872	2	1574.607	1	2	3
0.877033	1	0.908807	1	330.7377	2	2	3
1.750395	2	1.503806	2	759.5412	1	2	3
2.361581	2	1.904497	2	234.1133	2	1	3
2.930158	2	1.680812	2	3935.508	1	1	3
2.317685	2	1.313896	2	12547.59	1	2	3
1.834876	2	8.361808	2	2725.383	1	2	3
1.134872	2	1.096589	2	190.5273	2	2	2.666667
1.580153	2	2.002899	2	1603.28	1	2	6
0.848864	1	2.018748	2	296.0043	2	2	5.666667
0.945938	1	1.368339	2	72.27924	2	2	4.666667
1.869166	2	1.583949	2	672.4546	1	1	4.333333
2.621346	2	1.347671	2	1864.938	1	1	4
2.678534	2	1.937136	2	199.9708	2	2	4

1.360629	2	1.820455	2	606.4439	2	2	3.666667
2.454041	2	0.79534	1	349.0069	2	2	3.666667
2.636474	2	1.762919	2	229.5642	2	2	3
4.495871	2	2.251966	2	388.2238	2	2	3
2.500939	2	2.441038	2	280.3419	2	2	3
3.209328	2	0.908807	1	345.4713	2	2	3
4.721692	2	1.292627	2	230.7768	2	2	3
3.086847	2	1.892393	2	148.6877	2	1	3
4.575585	2	2.325829	2	319.1243	2	2	3
3.781109	2	1.726377	2	201.9875	2	2	5
3.284831	2	1.385472	2	1000.254	1	1	5
2.447657	2	4.354766	2	131.3567	2	2	3.666667
2.904989	2	1.099643	2	424.2483	2	2	3.666667
4.261167	2	2.778612	2	712.5185	1	1	3.666667
1.295273	2	1.968222	2	605.2538	2	2	3.666667
2.929903	2	1.047264	2	210.3815	2	2	3.333333
1.587441	2	1.782336	2	188.0316	2	1	3.333333
1.750395	2	1.503806	2	188.6976	2	2	3.333333
2.077444	2	1.938187	2	52.30441	2	2	3.333333
3.217461	2	1.210176	2	223.1928	2	2	3
1.294921	2	1.390278	2	753.3435	1	2	3
4.105044	2	1.049844	2	3901.296	1	2	3
3.277051	2	1.709904	2	222.5869	2	2	4
2.467715	2	1.16127	2	1126.678	1	2	3.666667
3.1927	2	1.456972	2	1682.093	1	1	3.666667
4.593696	2	2.064199	2	1886.05	1	2	3
4.688638	2	1.515807	2	440.3528	2	2	3
